# International Collaboration to Develop and Harmonize Drug Interaction Guidance for Nirmatrelvir/Ritonavir During COVID-19: Lessons Learned for Future Pandemic Preparedness

**DOI:** 10.1093/cid/ciaf606

**Published:** 2025-11-03

**Authors:** Safia Kuriakose, Alice Tseng, Sarita Boyd, Sara Gibbons, Justin Chiong, Fiona Marra, Alison Boyle, Tessa Senneker, Jomy George, Claire Lund, Pamela Belperio, Page Crew, Greg Eschenauer, Kimberly K Scarsi, Saye H Khoo, Alice Pau, Catia Marzolini, the LiON-PK collaboration, Melissa Badowski, Sarita Boyd, Alison Boyle, Jennifer Cocohoba, Justin Chiong, Sara Gibbons, Pierre Giguere, Saye H Khoo, Safia Kuriakose, Fiona Marra, Catia Marzolini, Salin Nhean, Alice Pau, Kimberly K Scarsi, Tessa Senneker, Kimberly Struble, Alice Tseng, Deborah Yoong, Page Crew, Jomy George

**Affiliations:** Clinical Research Directorate, Frederick National Laboratory for Cancer Research, Frederick, Maryland, USA; Toronto General Hospital, University Health Network and Leslie Dan Faculty of Pharmacy, University of Toronto, Toronto, Ontario, Canada; Division of Antivirals, Office of Infectious Diseases, Center for Drug Evaluation and Research, Food and Drug Administration, Silver Spring, Maryland, USA; Centre for Experimental Therapeutics, University of Liverpool, Liverpool, United Kingdom; Centre for Experimental Therapeutics, University of Liverpool, Liverpool, United Kingdom; Centre for Experimental Therapeutics, University of Liverpool, Liverpool, United Kingdom; Centre for Experimental Therapeutics, University of Liverpool, Liverpool, United Kingdom; Infection and Immunology Clinic, Kingston Health Sciences Centre, Kingston, Ontario, Canada; Division of Antivirals, Office of Infectious Diseases, Center for Drug Evaluation and Research, Food and Drug Administration, Silver Spring, Maryland, USA; Health, People and Human Services Group, ICF, Reston, Virginia, USA; Department of Veterans Affairs, Greater Los Angeles Health Care System, Los Angeles, California, USA; Division of Clinical Research, National Institute of Allergy and Infectious Diseases, National Institutes of Health, Bethesda, Maryland, USA; Department of Clinical Pharmacy, College of Pharmacy, University of Michigan, Ann Arbor, Michigan, USA; Department of Pharmacy Practice and Science, College of Pharmacy, University of Nebraska Medical Center, Omaha, Nebraska, USA; Centre for Experimental Therapeutics, University of Liverpool, Liverpool, United Kingdom; Division of Clinical Research, National Institute of Allergy and Infectious Diseases, National Institutes of Health, Bethesda, Maryland, USA; Centre for Experimental Therapeutics, University of Liverpool, Liverpool, United Kingdom; Service and Laboratory of Clinical Pharmacology, Department of Laboratory Medicine and Pathology, University Hospital Lausanne and University of Lausanne, Lausanne, Switzerland; Division of Infectious Diseases, Departments of Medicine and Clinical Research, University Hospital of Basel and University of Basel, Basel, Switzerland

**Keywords:** nirmatrelvir/ritonavir, drug interactions, COVID-19, international collaboration, pandemic

## Abstract

In December 2021 and January 2022, the severe acute respiratory syndrome coronavirus 2 (SARS-CoV-2) antiviral drug, nirmatrelvir/ritonavir, was authorized in the United States, Canada, and Europe. However, ritonavir has significant drug-drug interaction (DDI) potential, and information resources were incomplete or provided conflicting advice for certain DDIs. Within the challenging pandemic setting, nirmatrelvir/ritonavir was being prescribed largely by clinicians unfamiliar with ritonavir. Coronavirus disease 2019 (COVID-19) affects people with comorbid conditions and polypharmacy who could benefit from therapy, but prescriber uncertainty surrounding the appropriate management of DDIs was a barrier to the use of nirmatrelvir/ritonavir. To support clinicians, the National Institutes of Health (NIH) panel on COVID-19 guidelines, the Ontario COVID-19 Science Advisory Table, and the University of Liverpool independently developed prescribing guidelines. Ultimately, the groups united to establish the LiON-PK (Liverpool-Ontario-NIH Pharmacokinetics) collaboration. Here we describe how the team developed pragmatic and harmonized guidance for managing DDIs with nirmatrelvir/ritonavir. This framework may inform development of prescribing resources for other complex medications or for future pandemic preparedness.

## Teaser

In December 2021, the National Institutes of Health panel on coronavirus disease 2019 treatment guidelines, the Ontario Science Table, and the University of Liverpool collaborated to harmonize drug interaction guidance for nirmatrelvir/ritonavir. This article outlines the processes and lessons learned from this collaboration to inform future pandemic preparedness.

The coronavirus disease 2019 (COVID-19) pandemic stimulated unprecedented global collaboration of healthcare providers, academic researchers, and pharmaceutical companies to fight one of the fastest-spreading and deadliest infectious pathogens [1–3]. Healthcare organizations around the world began to develop guidance for practitioners struggling to manage this new disease that had no known treatment. In March 2020, the United States National Institutes of Health (NIH) convened a panel to write COVID-19 treatment guidelines [4]; After initially releasing a static drug-drug interaction (DDI) guidance in March 2020, the University of Liverpool (UoL) released a DDI online checker in May 2020 for drugs commonly prescribed for COVID-19 and other comedications [5]; in Canada, the Ontario COVID-19 Science Advisory Table (Ontario Science Table [OST]) [6] launched clinical practice guidelines in August 2020. These web-based guidance documents were designed as living documents to be updated/revised frequently as new research evidence warranted changes in recommendations.

On 22 December 2021, the US Food and Drug Administration (FDA) granted an emergency use authorization (EUA) to the first oral antiviral drug nirmatrelvir/ritonavir (Paxlovid; Pfizer), given as a 5-day course for patients with mild-to-moderate COVID-19 at risk of progression to severe disease. The timing of the EUA coincided with the emergence of the Omicron severe acute respiratory syndrome coronavirus 2 (SARS-CoV-2) variant, which led to surges in hospitalizations and deaths globally. There was a heightened need to make this new therapy available to as many eligible people as possible.

## DRUG INTERACTION CHALLENGES WHEN PRESCRIBING NIRMATRELVIR-RITONAVIR

A key challenge for prescribing this new antiviral drug was the inclusion of ritonavir, a potent inhibitor of cytochrome P450 (CYP) 3A4 enzyme and the drug transporter P-glycoprotein [7]. Ritonavir was approved in 1996 at a dose of 600 mg twice daily for the treatment of human immunodeficiency virus (HIV-1). While this high dose is no longer used for HIV treatment, ritonavir at daily doses of 100–200 mg is commonly used as a pharmacokinetic booster to increase concentrations of a concomitant active HIV protease inhibitor. Ritonavir has the potential to significantly increase concentrations of CYP3A4 and/or P-glycoprotein substrates, which may lead to toxic effects, especially for drugs with narrow therapeutic windows. In contrast, unlike the 600-mg twice-daily ritonavir treatment dose, the lower boosting dose does not result in a significant inhibition of CYP2D6 [8]. Ritonavir is also an inducer of certain CYP isoenzymes (CYP1A2, 2B6, 2C9, and 2C19) and uridine 5'-diphospho-glucuronosyltransferase and can reduce concentrations of drugs that are substrates of these pathways. However, the full inducing effect of ritonavir on drug metabolizing enzymes is not expected to be achieved during the 5-day course of nirmatrelvir/ritonavir.

In anticipation of the issuance of the nirmatrelvir/ritonavir EUA, clinical pharmacologists/pharmacists from the UoL, NIH, and OST teams simultaneously began preparing guidance documents. Their extensive experience in developing and releasing DDI guidance for ritonavir using similar methods allowed them to adapt and respond quickly in the pandemic setting (see Supplementary materials). These resources were designed to support busy clinicians, particularly those unfamiliar with managing ritonavir-associated DDIs who would be prescribing nirmatrelvir/ritonavir in high-risk patients. Furthermore, guidance was needed for other situations encountered in clinical practice and not covered in the initial EUA fact sheet (eg, dosing in renal impairment and administration in patients unable to swallow).

**Figure 1.**
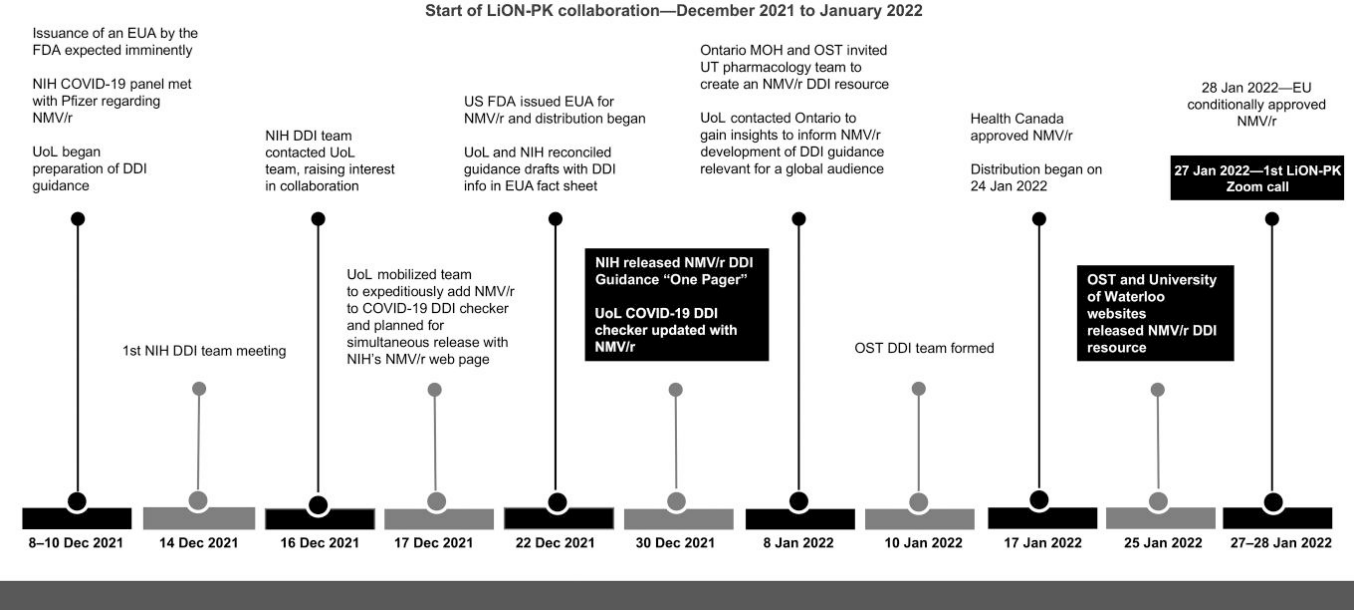
Timeline of events leading to the LiON-PK (Liverpool-Ontario-NIH Pharmacokinetics) collaboration. Abbreviations: DDI, drug-drug interaction; EU, European Union; EUA, emergency use authorization; FDA, Food and Drug Administration; MOH, Ministry of Health; NIH, National Institutes of Health; NMV/r, nirmatrelvir/ritonavir; OST, Ontario Science Table (Ontario COVID-19 Science Advisory Table); UoL, University of Liverpool; UT, University of Toronto.

Figure 1 illustrates the timeline for addition of nirmatrelvir/ritonavir DDI guidance to the UoL COVID-19 DDI checker and the NIH guidelines just 8 days after the FDA issuance of the EUA and well in advance of European Medicines Agency authorization in late January 2022. OST released its guidance in January 2022, shortly after the approval of nirmatrelvir/ritonavir by Health Canada.

Lack of data to support DDI recommendations for a 5-day course instead of long-term use of ritonavir was compounded by misinterpretation of the DDI risk in regulatory documents. For example, ritonavir-associated enzyme induction was initially mentioned in the European Medicines Agency and Health Canada monographs even though it is not relevant with the 5-day treatment course, and caution when prescribing antidepressants was mentioned, even though low-dose ritonavir only weakly inhibits CYP2D6.

## FORMATION OF COLLABORATION

The DDI challenges prompted the 3 groups to consult each other and establish the LiON-PK (Liverpool-Ontario-NIH Pharmacokinetics) collaboration. In January 2022, the group began monthly virtual teleconferencing and frequent communication/consultation via email to develop and update their respective guidances in a timely manner. The collaboration discussed antiviral pharmacology issues and challenges within the evolving global landscape. In the subsequent sections, we discuss steps taken by the teams to provide pragmatic, harmonized and up-to-date guidance for managing DDIs with nirmatrelvir/ritonavir: (1) collaboration with regulatory agencies; (2) harmonization of recommendations; (3) development of pragmatic recommendations and partnering with clinicians; (4) dissemination of guidance; and (5) responsiveness to prescribers’ needs and updates. This framework may inform development of prescribing resources for other commonly used complex medications or for future pandemic preparedness.

## COLLABORATION WITH REGULATORY AGENCIES

To facilitate the development and harmonization of recommendations, it is crucial for developers of DDI guidance to collaborate early on with regulatory agencies. A representative from the US FDA, a pharmacology consultant to the NIH COVID-19 treatment guidelines panel, participated in the LiON-PK collaboration, which allowed for mutual exchange of DDI information to quickly harmonize-improve recommendations in the nirmatrelvir/ritonavir EUA fact sheet for healthcare providers (fact sheet) and other DDI resources. LiON-PK members working in clinical practice settings provided viewpoints and experiences that could be relayed to the FDA nirmatrelvir/ritonavir review team for internal regulatory consideration. Similarly, the FDA provided insight into the general approach to DDI recommendations, including the importance of maintaining consistency between the nirmatrelvir/ritonavir fact sheet and FDA-approved labels for comedications and other clinical considerations.

Neither the nirmatrelvir/ritonavir fact sheet nor other DDI resources can include every possible DDI. However, other DDI resources have greater capacity to provide a more comprehensive list of comedications and clinical recommendations. The LiON-PK collaboration initially led to the inclusion of wide-ranging DDI information in the NIH guidelines; in turn, the FDA leveraged this information to add important DDIs (eg, apixaban, darifenacin, diazepam, saxagliptin, and zolpidem) to the nirmatrelvir/ritonavir fact sheet [9], given that these recommendations were consistent with approved labels for the concomitant drugs.

In other instances, the team agreed on recommendations that differed from the nirmatrelvir/ritonavir fact sheet. For example, the fact sheet recommended therapeutic drug monitoring (TDM) for tacrolimus, stating that nirmatrelvir/ritonavir use should be avoided when TDM of tacrolimus was not feasible. In contrast, the initial version of the NIH guidelines recommended alternative COVID-19 therapy for patients receiving tacrolimus. The fact sheet was less restrictive because TDM could be reasonably achieved in certain instances—for example, in high-risk patients diagnosed with COVID-19 while hospitalized for reasons other than COVID-19. The restrictive nature of the NIH guidelines was most relevant to an outpatient setting, where TDM was impractical. Following discussions about emerging clinical experience with tacrolimus and nirmatrelvir/ritonavir [10], the NIH guidelines were updated with recommendations more in line with the fact sheet. Even in instances where recommendations differed between the fact sheet and other resources, communication within the group about these differences allowed for mutual understanding of the rationale for discrepancies, the appropriate application of each recommendation, and attempts to better align recommendations, when possible.

## HARMONIZATION OF RECOMMENDATIONS

The primary source of DDI information for a newly approved or authorized drug is the product information, which refers to the product labeling, fact sheet, or monograph authorized by the relevant regulatory agency for each region or country. Many DDI resources are based on this information. However, when nirmatrelvir/ritonavir was first introduced in late 2021, product information was not uniformly consistent between countries (eg, for the number of drugs addressed or DDI interpretations). The LiON-PK team methodically compared labeling between countries and carefully evaluated discrepancies, which helped to harmonize recommendations. To factor in lower ritonavir dosing and shorter treatment duration, some recommendations differed from product information (Table 1); specific examples are provided in Supplementary Table 1.

**Table 1.** Description, Drug-Drug Interaction (DDI) Content, and Usage Metrics of the DDI Guidance From the University of Liverpool, the National Institutes of Health, and the Ontario COVID-19 Science Advisory Table

Guidance Characteristics	Guidance Source		
	UoL	NIH	OST
Writing group composition	Physicians and pharmacists with expertise in COVID-19, HIV, and hepatitis; PK/PD; modeling	10 Pharmacists with expertise in COVID-19, critical care, HIV, and ID; PK/PD; US regulations (1 FDA member)	13 Pharmacists with expertise in antithrombotic therapy, emergency medicine, family medicine, HIV/ID, oncology, psychiatry, and transplantation
DDI guide description	Web-based interactive DDI checker	Web page/PDF (outpatient focus)	Web page/PDF (DDI table and DOAC algorithm)
Comparison of DDI guidance documents with product information^a^			
Initial documents			
No. of drugs in guidance vs product information	558 (UoL) vs 137 (EMA product label/FDA EUA FS)	68 (NIH) vs 100 (FDA EUA FS)	133 (OST) vs 120 ( Health Canada monograph)
Portion of recommendations different, %	27	33	29
December 2022 documents			
No. of drugs in DDI guidance vs product information	880 (UoL) vs 177 (EMA product label/FDA EUA FS)	214 (NIH) vs 143 (FDA EUA FS)	139 (OST) vs 130 (Health Canada monograph)
Portion of recommendations different, %	25	29	32
DDI guidance revisions and usage metrics			
No. of revisions to drug entries in 2022^b^	143	37	73
No. of revisions with upgrades or downgrades in DDI severity	6 Upgrades; 44 downgrades	3 Upgrades; 23 downgrades	6 Upgrades; 67 downgrades
NMV/r DDI web page usage metrics for 2022	7 082 819 NMV/r queries (85% of all DDI queries); NMV/r DDI queries by country: US, 3 499 795 (49%); Canada, 549 151 (8%); UK, 442 677 (6%)	1 757 875 Page views (11% of all views on website); most viewed page in 2022; referrals to other DDI resources: UoL, 161 478; FDA, 47 045; OST, 37 618	244 695 Page views (43% of all views on website); most viewed page in 2022

Abbreviations: COVID-19, coronavirus disease 2019; DDI, drug-drug interaction; DOAC, direct-acting oral anticoagulant; EMA, European Medicines Agency; EUA FS, emergency use authorization fact sheet for healthcare providers; FDA, US Food and Drug Administration; HIV, human immunodeficiency virus; ID, infectious diseases; NIH, National Institutes of Health; NMV/r, nirmatrelvir/ritonavir; OST, Ontario Science Table (Ontario COVID-19 Science Advisory Table); PK/PD, pharmacokinetics/pharmacodynamics; UoL, University of Liverpool.

^a^Product information refers to the product labeling, fact sheet, or monograph authorized by the relevant regulatory agency for each region or country. Product information was not uniformly consistent between regions or countries (eg, for the number of drugs addressed or certain DDI interpretations).

^b^Includes revisions that did not lead to changes in DDI recommendations, such as updates to evidence summaries or comments.

A major benefit of the collaboration was harmonizing recommendations as much as possible to minimize confusion among prescribers. The team, new tracked, shared, and evaluated nirmatrelvir/ritonavir DDI literature together. DDI interpretations and recommendations were reviewed and discussed, particularly those identified as discordant. A consensus was generally reached during the LiON-PK meetings, which would then be applied when updating respective guidance documents. Topics requiring the most discussion included anticoagulants, calcium channel blockers (CCBs), contraception, and immunosuppressants. Other topics included the duration of significant CYP3A4 inhibition after the last dose of nirmatrelvir/ritonavir, appropriate timing for restarting or readjusting dosing of comedications, and optimizing management strategies in the context of balancing the benefits of nirmatrelvir/ritonavir treatment versus the risk of harm due to potentially severe DDIs.

## DEVELOPMENT OF PRAGMATIC RECOMMENDATIONS AND PARTNERING WITH CLINICIANS

Development of pragmatic and clinically suitable recommendations requires an interdisciplinary collaborative approach focused on patient outcomes. The LiON-PK team recognized that the responsibility of DDI management would often fall to frontline clinicians and pharmacists, many of whom had limited experience in managing ritonavir-associated DDIs. Some clinical sites did not have clinical pharmacists or resources available, and the need to initiate nirmatrelvir/ritonavir within 5 days of symptom onset limited the opportunity for clinicians to consult specialists/experts on DDI management.

The team collaborated with experts in other relevant therapeutic areas and considered the feasibility of DDI management strategies in a pandemic setting, where social distancing restrictions and reduced in-person staffing limited the ability for close clinical monitoring or laboratory monitoring (eg, TDM). Timely access to primary prescribers was also a challenge if new prescriptions were required for drug dosing changes or substitutions to manage DDIs.

Recommendations on DDI management were developed with a focus on fostering consensus in decision making and cooperative problem solving. The overarching goal was to provide straightforward, pragmatic advice to allow for safe use of nirmatrelvir/ritonavir, with minimal disruption/inconvenience to providers and/or patients. Supplementary Table 2 lists challenges encountered when developing guidance for general practicing clinicians. Comedications initially chosen for inclusion in DDI guidance documents were selected according to the prevalence of use among patients with comorbid conditions most likely to be at risk of severe COVID-19 outcomes (hence most likely to benefit from nirmatrelvir/ritonavir) and those with the greatest potential for high-risk DDIs with nirmatrelvir/ritonavir.

Once DDI management resources were disseminated, the team partnered with practitioners in the community and front lines to ensure that DDI guidance was clear and easily implemented. The team also maintained close contact with these practitioners to address specific concerns or evolving needs. Identified gaps or challenges were discussed at the LiON-PK meetings, and new information or updated recommendations were developed as needed. For instance, when managing DDIs with direct-acting oral anticoagulants was identified as a time-consuming challenge for community providers, a 2-page infographic stepwise algorithm to manage direct-acting oral anticoagulant DDIs was created by the OST group, with management information consistent with guidance in NIH and UoL resources (Supplementary materials).

Another example involved the interaction between CCBs and nirmatrelvir/ritonavir. Based on available data and experience, a 50% reduction in CCB dose was initially recommended. However, users often reported difficulties obtaining a new CCB prescription in time due to logistical barriers during the pandemic. Therefore, additional recommendations were developed to dose CCBs every other day or continue with the usual dose if the risk of bradycardia or hypotension was low.

## DISSEMINATION OF GUIDANCE DOCUMENTS

Given that nirmatrelvir/ritonavir was being prescribed by a wide range of practitioners without familiarity with ritonavir's drug characteristics [11], it was critical to increase awareness of the potential for nirmatrelvir/ritonavir DDIs and management strategies to avoid adverse outcomes. Furthermore, the off-label prescribing of nirmatrelvir/ritonavir beyond 5 days in select immunocompromised patients necessitated prescriber education regarding the impact of treatment duration on DDI potential. As nirmatrelvir/ritonavir was authorized only for a 5-day duration, differences in DDI potential based on duration of therapy were not addressed in regulatory labeling.

Given the global impact of COVID-19, as expected, user use of the DDI guidance documents was not limited to within-country resources. The NIH website on COVID-19 treatment guidelines served as a major anchor for prescriber information during the pandemic. A dedicated web page was developed to address nirmatrelvir/ritonavir DDIs, which linked to the OST DDI guidance documents, the UoL's web-based interactive COVID-19 DDI checker, and the FDA’s screening checklist tool to determine patients’ eligibility for nirmatrelvir/ritonavir. Creating a dedicated page to be accessed directly by providers or linked into other resources (including COVID-19 treatment guidance issued by government health authorities and/or medical societies) allowed broad and rapid dissemination of the DDI guidance.

**Figure 2.**
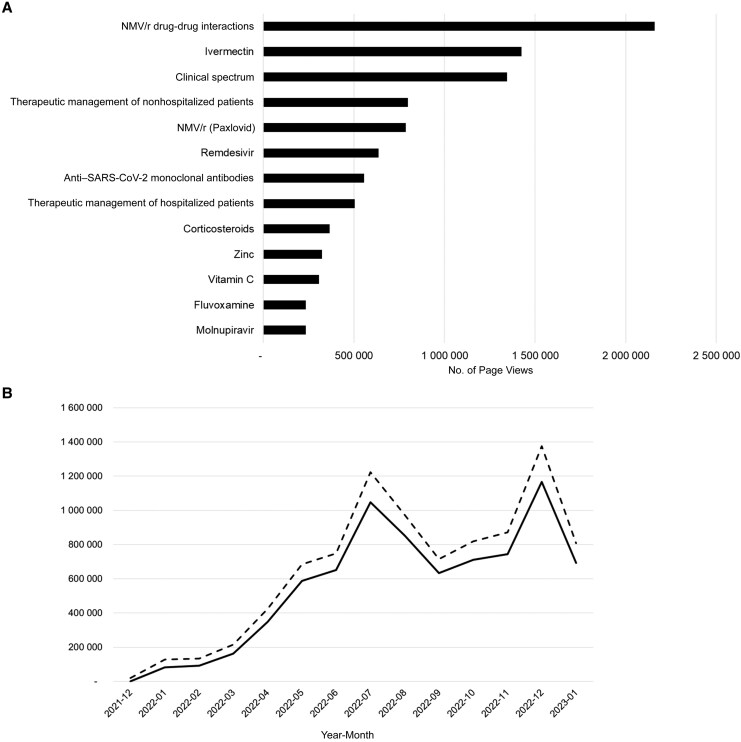
*A*, Top page views in the National Institutes of Health coronavirus disease 2019 (COVID-19) treatment guidelines from 30 December 2021 to 31 July 2023. Abbreviation: SARS-CoV-2, severe acute respiratory syndrome coronavirus 2. *B*, nirmatrelvir/ritonavir (NMV/r) drug-drug interaction queries relative to all other COVID-19 therapies (continuous line, NMV/r; dashed line, other COVID-19 drugs) for the University of Liverpool website. Numbers on x-axis represent year and month (ie, 2021-12 indicates December 2021; 2022-01, January 2022, etc.).

In 2022, NIH's nirmatrelvir/ritonavir DDI web page was the most viewed page of the NIH guidelines (Figure 2*A*), OST DDI guidance was the most accessed page on the OST site, and the UoL COVID-19 DDI checker fielded >7 million nirmatrelvir/ritonavir DDI queries with >1 million DDI queries in July and December 2022, coinciding with the summer and winter COVID-19 peaks (Figure 2*B*). NIH web statistics indicated cross-utilization of the UoL and OST resources (Table 1). Furthermore, almost 50% of queries to the UoL DDI checker came from US users. Overall, nirmatrelvir/ritonavir DDI guidance was highly accessed from each of these resources, indicating the demand and necessity for such guidance during the pandemic.

## RESPONSIVENESS TO PRESCRIBERS’ NEEDS AND UPDATES

The LiON-PK team strived to expand clinicians’ ability to prescribe nirmatrelvir/ritonavir by increasing the list of relevant comedications in DDI guidelines. To best respond to the needs of users, the UoL team conducted an online survey on their website from April to August 2022. Users were asked whether they found all the comedications they were looking for; if not, they were invited to submit new drug requests. Among the 19 000 received responses from 104 countries or territories, comedications were “found” in 70% of the responses. Among the “not found” responses, 1678 were valid drug requests not on the UoL website representing 422 new drugs; these requests were prioritized according to the frequency and/or clinical need. Drugs were added to the website and, by the end of 2024, all drugs with ≥15 requests were added (n = 140). Supplementary Table 3 provides the timeline of the responsiveness to users’ drug requests. In additionally, 86 drugs were identified from other sources (eg, drug requests by email) and added to the UoL site.

**Figure 3.**
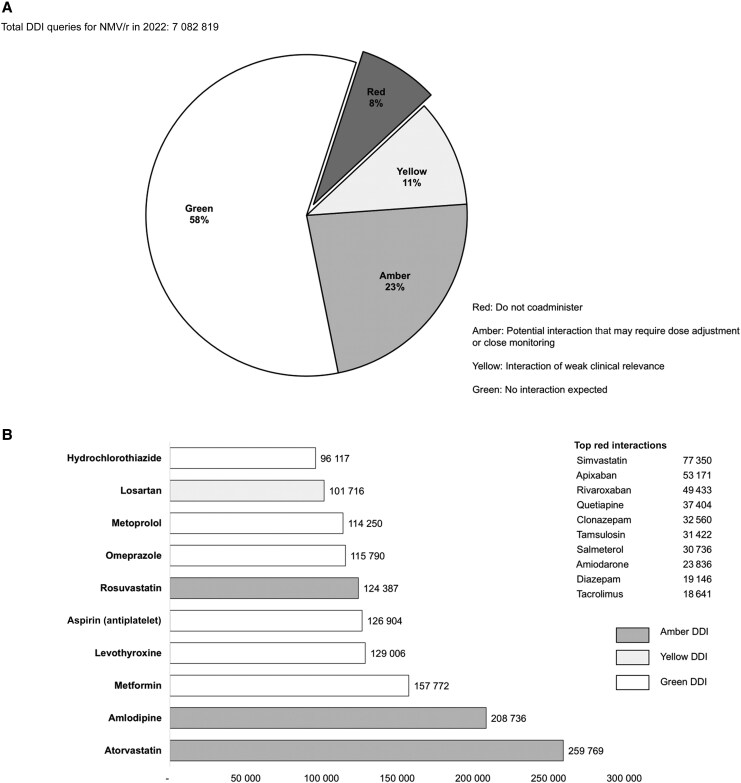
*A*, nirmatrelvir/ritonavir (NMV/r) drug-drug interaction (DDI) queries in 2022 by severity on the University of Liverpool website. *B*, Top comedications searches for 2022 on the University of Liverpool website.

Sharing the UoL website users’ statistics on nirmatrelvir/ritonavir DDIs queries (Figure 3*A*) and top searched comedications (Figure 3*B*) at the monthly LiON-PK meetings allowed the group to prioritize key drugs and respond to the needs of users either by creating extra resources or by refining guidance as new data emerged in the literature. For instance, the interaction with tacrolimus was initially labeled as “do not coadminister” following reports of nephrotoxicity [12]. This interaction was subsequently downgraded to “potential interaction manageable by dose adjustment and close monitoring” following the publication of a protocol which demonstrated the safe use of nirmatrelvir/ritonavir in kidney transplant recipients as supported by real-life data [13]. Regular review of the literature allowed for revision of recommendations as needed. In 2022, a total of 143 DDIs were revised on the UoL website, resulting in upgrades in severity for 6 DDIs and downgrades in 44 (Table 1).

Finally, requests from users identified prescribing gaps that were addressed in the framework of a research project. A frequent query was when to restart paused comedications or resume original doses of comedications, knowing that ritonavir causes an irreversible inhibition of CYP3A4 that does not resolve immediately after stopping ritonavir as it requires de novo enzyme synthesis to restore baseline metabolic activity [7]. The UoL team investigated the resolution of CYP3A4 inhibition after stopping ritonavir, using physiologically based pharmacokinetic modeling, which suggested that CYP3A4 inhibition was reduced by 80% after 48 hours in adults aged 20–50 years and after 72 hours in those aged ≥60 years. This led to the pragmatic recommendation to wait 2–3 days after completion of nirmatrelvir/ritonavir treatment before reinitiating paused comedications or readjusting their doses [14]. Key steps to inform prescribing of complex medications for future pandemic preparedness are listed in Table 2.

**Table 2.** Key Steps to Inform Prescribing of Complex Medications for Future Pandemic Preparedness

Collaboration with regulatory agencies Provide expert opinion on the management of drug-drug interactions based on clinical practice for consideration in product label Report prescribing discrepancies across labels
Harmonization of recommendations Methodically compare prescribing information across labels Discuss discrepancies during team meetings and reach a consensus to apply when developing the guidance
Development of pragmatic recommendations and partnering with clinicians Collaborate with experts on relevant comorbid conditions to develop clinically suitable recommendations Provide straightforward, pragmatic, actionable advice to allow safe prescribing with minimal disruption/inconvenience to providers and/or patients Prioritize medications selected according to the prevalence of use or greatest potential for harm because of underexposure or overexposure Maintain close contact with frontline prescribers and pharmacists to address specific concerns or evolving needs
Dissemination of the guidance Develop a dedicated website or web page to be accessed directly by providers Provide links to other credible drug-drug interaction sources and to guidance issued by government health authorities and/or medical societies
Responsiveness to prescribers’ needs and updates Conduct online survey >3 mo after release of the guidance to identify unmet needs Extract website user statistics to focus on needs by creating extra resources or refining guidance as new data emerge Perform regular review of the literature to provide the most up-to-date guidance Conduct research projects to address prescribing gaps

## ONGOING RELEVANCE OF THE COLLABORATIVE FRAMEWORK

After the urgency of the COVID-19 pandemic subsided, LiON-PK continued to meet regularly to discuss pharmacology issues of relevance related to HIV and other infectious diseases, such as discerning potentially clinically significant DDIs with tecovirimat during the mpox pandemic, and identifying gaps in knowledge regarding DDIs and the safety/pharmacokinetics of long-acting antiretrovirals in key populations including pregnancy, or those with concomitant illnesses, such as tuberculosis. This collaboration continues to help members refine and harmonize their drug information resources for non–COVID-19 conditions.

## ADDITIONAL CONSIDERATIONS

On reflection, there are areas where the team could have modified its approach. Subject matter experts for particularly challenging DDIs could have been engaged more proactively. In the future, specialists for commonly encountered comorbid conditions should be approached earlier to establish treatment guidance that can be disseminated quickly. For example, earlier collaboration with transplant experts could have helped to improve awareness regarding the major DDI between nirmatrelvir/ritonavir and tacrolimus. In addition, the group could have liaised earlier with the manufacturer to discuss important prescribing gaps (eg, dosing in renal impairment).

## CONCLUSIONS

Outbreaks of emerging infections disproportionately affect people with preexisting comorbid conditions, where harms arising from DDIs can be missed, or misattributed to a new disease not yet fully understood. The urgency and need for new treatments mean that major uncertainties remain even after EUA. These key gaps can sometimes prevent the use of drugs in those who are at greatest risk from severe disease, where the risk from DDIs needs to be weighed against the consequences of not having effective treatment. We have shown here that a broad international consensus can be reached to harmonize recommendations on a risk-proportionate deployment of new drugs, even when regulatory labeling does not completely align. This approach, used for nirmatrelvir/ritonavir guidance decisions, weighs the likelihood of harms from short-course therapy, the ability to mitigate or monitor those harms, the consequences of denying a particular antiviral and the availability of alternative treatments. Finally, the infrastructure created, including the framework for regular collaborative meetings and the platforms for disseminating prescribing guidance and DDI tools, should be maintained and developed in “peacetime” ahead of any future pandemic.

## Supplementary Material

ciaf606_Supplementary_Data

## Supplementary Data

Supplementary materials are available at *Clinical Infectious Diseases* online. Consisting of data provided by the authors to benefit the reader, the posted materials are not copyedited and are the sole responsibility of the authors, so questions or comments should be addressed to the corresponding author.
