# Supplementary material for: International Collaboration to Develop and Harmonize Drug Interaction Guidance for Nirmatrelvir/Ritonavir During COVID-19: Lessons Learned for Future Pandemic Preparedness
Source: Clin Infect Dis. 2025 Nov 3;82(3):e446–54. doi: 10.1093/cid/ciaf606 (PMC13017396; doi:10.1093/cid/ciaf606)
Supplement: ciaf606_Supplementary_Data [file ciaf606_supplementary_data.pdf]

Supplementary Materials: International Collaboration to Develop and Harmonize Drug Interaction Guidance for Nirmatrelvir/Ritonavir During COVID-19: Lessons Learned for Future Pandemic Preparedness

Table of contents

Supplementary Table 1: Examples of drug interaction recommendations that differed between nirmatrelvir/ritonavir product information and the consensus & guidance reached by the LiON-PK team…………………………………..……………………….....p2

Supplementary Table 2: Challenges when developing guidance for general practicing clinicians………………………………………………………………………………………...p4

Supplementary Table 3: Responsiveness to drug requests from users……..........................p5

NIH COVID-19 Treatment Guidelines: Drug-Drug Interactions Between Ritonavir-Boosted Nirmatrelvir (Paxlovid) and Concomitant Medications…………………………………...….p6

University Health Network: Management of Nirmatrelvir/Ritonavir (Paxlovid^{TM}) Drug-Drug Interactions in Oncology……………………………………………………….……………p13

Ontario Science Table: Nirmatrelvir/Ritonavir (Paxlovid^{TM}) What Prescribers and Pharmacists Need to Know………………..……………………………………………………..p33

Ontario Science Table: Paxlovid for a Patient on a DOAC..p51

Supplementary Materials

Table S1: Examples of drug interaction recommendations that differed between nirmatrelvir/ritonavir product information and the consensus & guidance reached by the LiON-PK team. The mechanism by which ritonavir may cause the interaction is described in brackets.

**Comedications**	**Mechanism DDI**	**Product information**[a]	**LiON-PK consensus**
Amlodipine	↑ amlodipine<br>(CYP3A4 inhibition)	Use with caution, dose reduction may be needed.	More detailed recommendation provided.<br>⇨ "Reduce dose by 50% or take every other day. Resume usual dose 2-3 days after completing NMV/r. May consider maintaining current dosing in patients at low risk of bradycardia or hypotension".
Antidepressants	↑ antidepressant<br>(CYP2D6 inhibition)	Use with caution, dose reduction of antidepressants may be needed.	CYP2D6 inhibition is not clinically meaningful at low ritonavir dose.<br>⇨ DDI downgraded with advice "no dose adjustment needed".
Corticosteroids	↑ corticosteroid<br>(CYP3A4 inhibition)	Risk for Cushing's syndrome and adrenal suppression. Use of corticosteroids restricted to beclomethasone and prednisolone.	Risk of Cushing's syndrome considered to be low with the short treatment duration. ⇨ DDI downgraded to "no interaction expected".
Bupropion	↓ bupropion<br>(CYP2B6 induction)	Reduced bupropion concentration. Monitor clinical response.	CYP2B6 induction not relevant with short NMV/r treatment course.<br>⇨ DDI downgraded with advice "no dose adjustment needed".
Ethinyl estradiol (EE)	↓ EE<br>(UGT induction)	Consider EE dosage increase/alternate contraceptive measures. Use an additional nonhormonal method of contraception during NMV/r treatment and for one menstrual cycle afterwards.	UGT induction not relevant with short NMV/r treatment course. Contraception efficacy is related to the progesterone component of combined hormonal contraceptives which is increased by ritonavir. ⇨ DDI downgraded with advice "continue with standard dosing".

Levothyroxine	↓ levothyroxine (UGT induction)	Monitor thyroid-stimulating hormone at least the first month after starting and/or ending NMV/r treatment.	UGT induction not relevant with short NMV/r treatment course. ⇨ DDI downgraded with advice "no dose adjustment needed".
Methadone	↓ methadone (CYP2B6 induction)	Increased methadone dose may be necessary based on clinical response.	Moderate-weak decreases in methadone exposure have been reported with chronic use of ritonavir as booster. ⇨ DDI downgraded with advice "no dose adjustment a priori needed".
**Comedications**	**Mechanism DDI**	**Product information[a]**	**LiON-PK consensus**
Tamsulosin	↑ tamsulosin (CYP3A4 inhibition)	Avoid concomitant use.	NMV/r is expected to increase tamsulosin exposure by 3-fold (based on DDI with ketoconazole another strong CYP3A4 inhibitor). However, DDI is manageable (large experience with people living with HIV on ritonavir boosted protease inhibitor).<br>⇨ DDI downgraded with advice "tamsulosin dose should not exceed 0.4 mg/day. Monitor for hypotension".

DDI = drug-drug interaction; NMV/r = nirmatrelvir/ritonavir. a = Product information refers to the product labeling, fact sheet, or monograph authorized by the relevant regulatory agency for each region or country. Product information was not uniformly consistent between regions or countries.

Table S2: Challenges when developing guidance for general practicing clinicians.

- HIV healthcare providers are familiar with DDIs associated with ritonavir, but this is not the case for general practitioners.
- Patients at risk for severe COVID-19 are generally older, people who are immunocompromised (such as patients with a history of organ transplant or patients undergoing cancer therapies), or people with comorbidities (such as cardiovascular, pulmonary, renal or liver diseases, diabetes). Most of them have polypharmacy which may interact with ritonavir.
- For treatment of HIV, ritonavir is given as daily therapy indefinitely, whereas ritonavir-boosted nirmatrelvir is only used for a 5-day course. The pharmacokinetic impact of 5 days of enzyme inhibition is not well studied and not known.
- Chronic ritonavir causes induction of CYP1A2, CYP2C9, CYP2C19 and UGT [7], but this is not the case with the short 5 days treatment course thus DDI guidance for ritonavir-boosted HIV protease inhibitors may not necessarily be applicable to nirmatrelvir/ritonavir.
- Clinical management of comedications may vary, depending on the extent of the interaction, the half-life of the comedication, the therapeutic window of the comedication, and the indication of the comedication.
- Implementing dose adjustments or alternative medications remained challenging due to the short nirmatrelvir/ritonavir treatment course, getting timely responses from specialist or GP prescribers, and other logistical challenges during the pandemic.
- Some comedication may result in significant untoward consequences – e.g. immunosuppressants, anti-arrhythmic drugs, DOACs, cancer chemotherapy, etc. which warrants a thorough evaluation of the risk-benefit of prescribing nirmatrelvir/ritonavir.
- Some comedications can be withheld while on nirmatrelvir/ritonavir treatment, others may need dosage reduction, others cannot be withheld.
- During the pandemic monitoring was difficult (e.g. lab monitoring, side effects assessment) as patients with COVID-19 should be isolated.
- Guidance needs to be practical and actionable.

Table S3: Responsiveness to drug requests from users.

Year	Quarter	Requests addressed	Requests remaining	% addressed
Total drug requests received			1678	
**2023**		**965**	**713**	**57.5**
	2023 Q1	342	1336	20.4
	2023 Q2	136	1200	28.5
	2023 Q3	249	951	43.3
	2023 Q4	238	713	57.5
**2024**		**99**	**614**	**63.4**
	2024 Q1	47	666	60.3
	2024 Q2	8	658	60.8
	2024 Q3	37	621	63.0
	2024 Q4	7	614	63.4

Drug-Drug Interactions Between Ritonavir-Boosted Nirmatrelvir (Paxlovid) and Concomitant Medications

Last Updated: February 29, 2024

Ritonavir, a strong cytochrome P450 (CYP) 3A4 inhibitor and a P-glycoprotein (P-gp) inhibitor, is coadministered with nirmatrelvir to increase the blood concentration of nirmatrelvir, thereby making it effective against SARS-CoV-2. Ritonavir may also increase blood concentrations of certain concomitant medications. The Food and Drug Administration (FDA) prescribing information includes a boxed warning about significant drug-drug interactions between ritonavir-boosted nirmatrelvir (Paxlovid) and other medications.

Before prescribing ritonavir-boosted nirmatrelvir to treat patients with mild to moderate COVID-19, carefully review the patient's concomitant medications, including over-the-counter medicines, herbal supplements, and recreational drugs. Clinicians should consider the potential benefits of treatment with ritonavir-boosted nirmatrelvir, the potential risks of drug-drug interactions, and whether any risks related to drug-drug interactions can be safely managed. Clinicians should be aware that many commonly used medications can be safely coadministered with ritonavir-boosted nirmatrelvir despite its drug-drug interaction potential. Box 1 includes commonly prescribed medications that are not expected to have clinically relevant interactions with ritonavir-boosted nirmatrelvir.

Because ritonavir-boosted nirmatrelvir is the only highly effective oral antiviral for the treatment of COVID-19, drug interactions that can be safely managed should not preclude the use of this medication.

Box 1. Select Outpatient Medications Not Expected to Have Clinically Relevant Interactions With Ritonavir-Boosted Nirmatrelvir (Paxlovid)

This list is primarily based on the most common medication searches by U.S. users on the Liverpool COVID-19 Drug Interactions website.

Medications Without Clinically Relevant Interactions

These medications do not require dose adjustments when coadministered with ritonavir-boosted nirmatrelvir, and the patients do not require additional monitoring. This list does not include all the noninteracting medications within each drug category.

Acid Reducers
- Famotidine
- Omeprazole
- Pantoprazole

Allergy
- Cetirizine
- Diphenhydramine
- Fexofenadine
- Loratadine

Anti-Infectives
- Azithromycin
- Cidofovir
- Hydroxychloroquine
- Tecovirimat
- Valacyclovir

Cardiovascular
- Aspirin
- Atenolol
- Carvedilol
- Furosemide
- Hydrochlorothiazide
- Irbesartan
- Isosorbide dinitrate
- Lisinopril
- Losartan
- Metoprolol
- Prasugrel

Diabetes
- Empagliflozin
- Insulin
- Metformin
- Pioglitazone

Immunosuppressants
- Abrocitinib
- Baricitinib
- Methotrexate
- Mycophenolate
- Prednisone

Lipid-Modifiers
- Ezetimibe
- Pitavastatin
- Pravastatin

Migraine
- Frovatriptan
- Naratriptan
- Rizatriptan
- Sumatriptan
- Zavegepant

Neuropsychiatric
- Amitriptyline
- Bupropion

Neuropsychiatric, cont'd
- Citalopram
- Duloxetine
- Escitalopram
- Fluoxetine
- Gabapentin
- Lorazepam
- Nortriptyline
- Olanzapine
- Paroxetine
- Sertraline
- Venlafaxine

Pain
- Acetaminophen
- Aspirin
- Codeine
- Ibuprofen
- Meloxicam
- Naproxen

Respiratory
- Corticosteroids (inhaled/nasal)
- Formoterol
- Montelukast

Miscellaneous
- Allopurinol
- Contraceptives (PO)[a]
- Cyclobenzaprine
- Donepezil
- Enoxaparin
- Finasteride
- Levothyroxine
- Most mAb products[b]
- Ondansetron

Medications Without Clinically Relevant Interactions, continued
[a] Coadministering contraceptive products that contain ethinyl estradiol with ritonavir-boosted nirmatrelvir may result in lower ethinyl estradiol concentrations. The FDA prescribing information for ritonavir-boosted nirmatrelvir suggests that individuals who use these types of contraceptive products should consider using an additional, nonhormonal contraceptive method. However, the lower ethinyl estradiol concentrations are not expected to be clinically significant during the 5 days of therapy. The progestin concentration of a combined hormonal contraceptive is expected to remain similar or increase with coadministration, which would maintain the effectiveness of the PO contraceptive.
[b] Ritonavir-boosted nirmatrelvir interacts with certain conjugated mAbs, such as ado-trastuzumab emtansine, mirvetuximab soravtansine, brentuximab vedotin, enfortumab vedotin, polatuzumab vedotin, and tisotumab vedotin. Before coadministering ritonavir-boosted nirmatrelvir and any of these conjugated mAbs, refer to the drug's FDA prescribing information and consult the patient's specialist providers as needed.
**Key:** FDA = Food and Drug Administration; mAb = monoclonal antibody; PO = oral

Medications That Have Clinically Relevant Drug-Drug Interactions With Ritonavir-Boosted Nirmatrelvir (Paxlovid)

Clinicians should be aware that, in some cases, drug-drug interactions with ritonavir-boosted nirmatrelvir may lead to serious or life-threatening drug toxicities. The recommended treatment course of ritonavir-boosted nirmatrelvir for COVID-19 is 5 days. CYP3A4 inhibition occurs rapidly, with maximum inhibition occurring within 48 hours of ritonavir initiation.[1] After treatment is completed and ritonavir is discontinued, 70% to 90% of CYP3A4 inhibition resolves within 2 to 3 days.[2] The time to resolution of inhibition varies based on factors such as the patient's age; therefore, resolution may take longer in some individuals, such as in adults of advanced age.

Ritonavir is also an inhibitor of CYP2D6, P-gp, and organic anion transporting polypeptide (OATP) 1B1. When used for longer durations or chronically, ritonavir may induce CYP1A2, CYP2B6, CYP2C8, CYP2C9, CYP2C19, and uridine diphosphate-glucuronyltransferase (UGT).

Nirmatrelvir and ritonavir are CYP3A4 substrates. Ritonavir-boosted nirmatrelvir should not be given within 2 weeks of administering a strong CYP3A4 inducer (e.g., St. John's wort, rifampin). Ritonavir-boosted nirmatrelvir is **contraindicated** in this setting because the delayed offset of enzyme induction may reduce the concentrations of nirmatrelvir and ritonavir, rendering the treatment ineffective against SARS-CoV-2. An alternative treatment for COVID-19 should be prescribed.

Identifying Drug-Drug Interactions

Consult the following resources for information on identifying and managing drug-drug interactions.

- Quick reference lists:
  - Box 1 above lists select outpatient medications that are not expected to have clinically relevant interactions with ritonavir-boosted nirmatrelvir.
  - Box 2 below lists select outpatient medications that have clinically relevant drug-drug interactions with ritonavir-boosted nirmatrelvir.
- Web-based drug-drug interaction checker:
  - The Liverpool COVID-19 Drug Interactions website
- Tables with guidance on managing specific drug-drug interactions:
  - The University of Waterloo/University of Toronto drug interaction guide for ritonavir-boosted nirmatrelvir
  - The FDA prescribing information for ritonavir-boosted nirmatrelvir

Management Strategies for Drug-Drug Interactions

Consider the magnitude and significance of the potential drug-drug interaction when choosing management strategies for patients who will be receiving ritonavir-boosted nirmatrelvir. Potential strategies include:

- Increasing monitoring for potential adverse effects to the concomitant medication.
- Adjusting the dose of the concomitant medication.
- Temporarily withholding the concomitant medication.
- Using an alternative to the concomitant medication.
- Using alternative COVID-19 therapies (see Therapeutic Management of Nonhospitalized Adults With COVID-19).

Use the chosen strategy for the 5-day treatment course of ritonavir-boosted nirmatrelvir and for at least 2 to 3 days after treatment completion. The strategy may need to continue for a longer duration if ritonavir-boosted nirmatrelvir is initiated in an adult of advanced age or if the interacting medication has a long half-life.

Consider consulting with an expert (e.g., a pharmacist or the patient's specialist providers) when treating patients who are receiving highly specialized therapies or drugs that are prone to concentration-dependent toxicities, such as certain anticonvulsant, anticoagulant, immunosuppressant, antiarrhythmic, chemotherapeutic, and neuropsychiatric drugs.

The decision to prescribe ritonavir-boosted nirmatrelvir to patients who are receiving calcineurin and mammalian target of rapamycin inhibitors should always be made in consultation with the patient's specialist providers. Among reports submitted to the FDA Adverse Event Reporting System, the most commonly reported concomitant medications that resulted in serious adverse reactions, including fatal events, were calcineurin inhibitors (e.g., tacrolimus).[3] Ritonavir-boosted nirmatrelvir may be prescribed to certain patients who are receiving these medications if an expert in managing the interaction is available and close therapeutic drug monitoring is logistically feasible. Otherwise, an alternative therapy for COVID-19 should be considered. See the American Society of Transplantation statement for more information.

Interactions between ritonavir-boosted nirmatrelvir and chemotherapeutic agents should also be managed in consultation with the patient's specialist providers. For guidance on managing these interactions, refer to the FDA prescribing information for ritonavir-boosted nirmatrelvir and the prescribing information for the chemotherapeutic agent. The University Health Network/Kingston Health Sciences Centre provides an additional resource for evaluating drug-drug interactions between ritonavir-boosted nirmatrelvir and chemotherapeutic agents.

Patients should be counseled about ritonavir-boosted nirmatrelvir's drug-drug interaction potential and the signs and symptoms of potential adverse effects. If ritonavir-boosted nirmatrelvir is prescribed to patients who take certain recreational drugs, those patients will require counseling and careful monitoring for adverse effects.

Box 2. Select Outpatient Medications That Have Clinically Relevant Drug-Drug Interactions With Ritonavir-Boosted Nirmatrelvir (Paxlovid)

The guidance in Box 2 is based on the drug-drug interaction potential of the FDA-approved, 5-day course of ritonavir-boosted nirmatrelvir.

Not all medications that may interact with ritonavir-boosted nirmatrelvir are included in Box 2. Deviation from the recommended strategies may be appropriate in certain clinical scenarios.

Prescribe Alternative COVID-19 Therapy			
For these medications, management strategies are not possible or feasible, or the risks outweigh the potential benefits.			
**Anticonvulsants**<br>• Carbamazepine<br>• Phenobarbital<br>• Phenytoin<br>• Primidone<br>**Anti-Infectives**<br>• Glecaprevir/pibrentasvir<br>• Rifampin<br>• Rifapentine<br>**Immunosuppressants**<br>• Voclosporin	**Cardiovascular**<br>• Amiodarone<br>• Clopidogrel[a,b]<br>• Disopyramide<br>• Dofetilide<br>• Dronedarone<br>• Eplerenone<br>• Flecainide<br>• Ivabradine<br>• Propafenone<br>• Quinidine	**Neuropsychiatric**<br>• Clozapine<br>• Lurasidone<br>• Midazolam (PO)<br>• Pimozide<br>**Pulmonary Hypertension**[c]<br>• Sildenafil<br>• Tadalafil<br>• Vardenafil	**Miscellaneous**<br>• Bosentan<br>• Certain chemotherapeutic agents[d]<br>• Ergot derivatives<br>• Lumacaftor/ivacaftor<br>• St. John's wort<br>• Tolvaptan

Temporarily Withhold Concomitant Medication, if Clinically Appropriate			
Withhold these medications during ritonavir-boosted nirmatrelvir (Paxlovid) treatment and for at least 2–3 days after treatment completion. They may need to be withheld for longer if the patient is an adult of advanced age or if the interacting medication has a long half-life. If withholding is not clinically appropriate, use an alternative concomitant medication or COVID-19 therapy.			
**Anticoagulants**<br>• Rivaroxaban[e]<br>**Anti-Infectives**<br>• Erythromycin<br>**BPH**<br>• Alfuzosin<br>• Silodosin<br>**Cardiovascular**<br>• Aliskiren<br>• Ranolazine<br>• Ticagrelor[b]<br>• Vorapaxar	**Immunosuppressants**[f]<br>• Everolimus<br>• Sirolimus<br>• Tacrolimus<br>**Lipid-modifiers**<br>• Atorvastatin[g]<br>• Lomitapide<br>• Lovastatin[g]<br>• Rosuvastatin[g]<br>• Simvastatin[g]	**Migraine**<br>• Eletriptan<br>• Rimegepant<br>• Ubrogepant<br>**Neuropsychiatric**<br>• Daridorexant<br>• Lemborexant<br>• Suvorexant<br>• Triazolam[h]<br>**Erectile Dysfunction**<br>• Avanafil	**Respiratory**<br>• Salmeterol<br>**Miscellaneous**<br>• Certain chemotherapeutic agents[d]<br>• Colchicine[i]<br>• Finerenone<br>• Flibanserin<br>• Naloxegol

Adjust Concomitant Medication Dose and Monitor for Adverse Effects			
Reduce the dose and/or extend the dosing interval of the concomitant medication. Consult the Liverpool COVID-19 Drug Interactions website or the University of Waterloo/University of Toronto drug interaction guide for specific dosing recommendations.[j] If the dose of the concomitant medication cannot be adjusted, withhold the medication (if clinically appropriate) or use an alternative concomitant medication or COVID-19 therapy.			
**Anticoagulants**<br>• Apixaban<br>• Dabigatran<br>• Edoxaban<br>**Anti-Infectives**<br>• Clarithromycin<br>• Itraconazole<br>• Ketoconazole<br>• Maraviroc<br>• Rifabutin<br>**BPH**<br>• Tamsulosin	**Cardiovascular**<br>• Amlodipine<br>• Cilostazol<br>• Digoxin<br>• Diltiazem<br>• Felodipine<br>• Nifedipine<br>• Verapamil<br>**Diabetes**<br>• Saxagliptin<br>**Erectile Dysfunction**[c]<br>• Sildenafil<br>• Tadalafil<br>• Vardenafil	**Immunosuppressants**<br>• Cyclosporine[f]<br>• Dexamethasone[k]<br>• Fedratinib<br>• Ruxolitinib<br>• Tofacitinib<br>• Upadacitinib<br>**Migraine**<br>• Almotriptan[i]<br>**Neuropsychiatric**<br>• Alprazolam[h]<br>• Aripiprazole<br>• Brexpiprazole	**Neuropsychiatric**, cont'd<br>• Buspirone<br>• Cariprazine<br>• Chlordiazepoxide[h]<br>• Clobazam[h]<br>• Clonazepam[h]<br>• Clorazepate[h]<br>• Diazepam[h]<br>• Estazolam[h]<br>• Flurazepam[h]<br>• Iloperidone<br>• Lumateperone<br>• Pimavanserin<br>• Quetiapine<br>• Trazodone

Adjust Concomitant Medication Dose and Monitor for Adverse Effects, continued			
**Pain**<br>• Fentanyl<br>• Hydrocodone<br>• Oxycodone<br>**Pulmonary Hypertension**<br>• Riociguat	**Miscellaneous**<br>• Certain chemotherapeutic agents[d]<br>• Darifenacin	**Miscellaneous**, cont'd<br>• Elexacaftor/tezacaftor/ivacaftor<br>• Eluxadoline<br>• Ivacaftor	**Miscellaneous**, cont'd<br>• Solifenacin<br>• Tezacaftor/ivacaftor

Continue Concomitant Medication and Monitor for Adverse Effects			
There is no need to pre-emptively adjust the doses of these drugs, but dose adjustments may be considered in patients with a high risk of AEs. Educate patients about potential AEs. Consult the Liverpool COVID-19 Drug Interactions website or the University of Waterloo/University of Toronto drug interaction guide for monitoring guidance and dose adjustment information.[j]			
**Anticoagulants**<br>• Warfarin<br>**Anti-Infectives**<br>• Brincidofovir[l]<br>• Cobicistat- or ritonavir-boosted ARV drugs<br>• Isavuconazole<br>• Posaconazole<br>• Voriconazole	**BPH**<br>• Doxazosin<br>• Terazosin<br>**Diabetes**<br>• Glyburide<br>**Cardiovascular**<br>• Mexiletine<br>• Sacubitril<br>• Valsartan	**Migraine**<br>• Zolmitriptan<br>**Neuropsychiatric**<br>• Haloperidol<br>• Hydroxyzine<br>• Mirtazapine<br>• Risperidone<br>• Ziprasidone<br>• Zolpidem	**Pain**<br>• Buprenorphine<br>• Hydromorphone<br>• Methadone<br>• Morphine<br>• Tramadol<br>**Miscellaneous**<br>• Certain chemotherapeutic agents[d]<br>• Certain conjugated mAbs[m]<br>• Oxybutynin

[a] Reduced effectiveness of clopidogrel is likely. It may be acceptable to continue using clopidogrel if the benefits of using ritonavir-boosted nirmatrelvir outweigh the risk of reduced clopidogrel effectiveness.

[b] For patients with a very high risk of thrombosis (e.g., those who received a coronary stent within the past 6 weeks), consider prescribing an alternative antiplatelet (e.g., prasugrel, if clinically appropriate) or an alternative COVID-19 therapy.

[c] Some PDE5 inhibitors are used to treat both PAH and erectile dysfunction; however, the doses used to treat PAH are higher than those used for erectile dysfunction. Because of this, and because PDE5 inhibitors are used chronically in patients with PAH, coadministration with ritonavir-boosted nirmatrelvir is **contraindicated** in these patients. PDE5 inhibitors can be coadministered with ritonavir-boosted nirmatrelvir in patients with erectile dysfunction, though the dose of the PDE5 inhibitor should be adjusted.

[d] Ritonavir-boosted nirmatrelvir may increase concentrations of some chemotherapeutic agents, leading to an increased potential for drug toxicities. Some chemotherapeutic agents may decrease the effectiveness of ritonavir-boosted nirmatrelvir. Please refer to the FDA prescribing information for ritonavir-boosted nirmatrelvir and the prescribing information for the chemotherapeutic agent and consult the patient's specialist provider. The University Health Network/Kingston Health Sciences Centre is an additional resource for evaluating drug-drug interactions for chemotherapeutic agents.

[e] For patients with a high risk of arterial or venous thrombosis (e.g., those who had a stroke within the past 3 months with a CHA_2DS_2-VASc score of 7–9 or a pulmonary embolism within the past month), consult the patient's primary or specialty provider and consider using an alternative anticoagulant (e.g., LMWH) or an alternative COVID-19 therapy. For patients with a lower risk of arterial or venous thrombosis, clinicians may consider administering low-dose aspirin while rivaroxaban is being withheld.

[f] The use of another COVID-19 therapy may need to be considered. These immunosuppressants have significant drug-drug interaction potential with ritonavir, and they **should not be used** if close monitoring, including therapeutic drug monitoring (i.e., measuring drug concentrations), is not feasible. Consult the patient's specialist providers before coadministering these immunosuppressants with ritonavir-boosted nirmatrelvir. See the American Society of Transplantation statement for more information.

[g] Withhold lovastatin and simvastatin for at least 12 hours before initiating ritonavir-boosted nirmatrelvir, during treatment, and for 5 days after treatment completion. Withhold atorvastatin and rosuvastatin at the beginning of treatment with ritonavir-boosted nirmatrelvir and resume after completing the 5-day course. If withholding a statin is not clinically appropriate (e.g., because the patient recently had a myocardial infarction), clinicians can reduce the doses of

Continue Concomitant Medication and Monitor for Adverse Effects, continued
atorvastatin and rosuvastatin and continue treatment. However, lovastatin and simvastatin should be switched to an alternative statin.

[h] The guidance on managing drug-drug interactions between certain benzodiazepines and ritonavir-boosted nirmatrelvir can vary significantly between product information resources. Note that abrupt discontinuation or rapid dose reduction of benzodiazepines may precipitate an acute withdrawal reaction.[4] The risk is greatest for patients who have been using high doses of benzodiazepines over an extended period.

[i] Do not coadminister this medication with ritonavir-boosted nirmatrelvir in patients with hepatic or renal impairment.

[j] For medications that are not included on the Liverpool COVID-19 Drug Interactions website or in the University of Waterloo/University of Toronto drug interaction guide, refer to the FDA labels for information on coadministering these medications with ritonavir or other strong CYP3A4 and/or P-gp inhibitors (e.g., ketoconazole).

[k] Dexamethasone exposure is expected to increase 2.60-fold when dexamethasone is coadministered with ritonavir-boosted nirmatrelvir.[5] Clinicians should weigh the risks and benefits of continuing the patient's normal dose of dexamethasone (while monitoring for AEs) against the risks and benefits of decreasing the dose. Patients who are receiving higher doses of dexamethasone will be at a greater risk of AEs.

[l] Patients should take ritonavir-boosted nirmatrelvir at least 3 hours after taking brincidofovir.

[m] Ritonavir-boosted nirmatrelvir interacts with certain conjugated mAbs, such as ado-trastuzumab emtansine, mirvetuximab soravtansine, brentuximab vedotin, enfortumab vedotin, polatuzumab vedotin, and tisotumab vedotin. Before coadministering ritonavir-boosted nirmatrelvir and any of these conjugated mAbs, refer to the drug's FDA prescribing information and consult the patient's specialist providers as needed.

Key: AE = adverse effect; ARV = antiretroviral; BPH = benign prostatic hyperplasia; CHA_2DS_2-VASc = congestive heart failure, hypertension, age, diabetes, stroke, vascular disease; CYP = cytochrome P450; FDA = Food and Drug Administration; LMWH = low-molecular-weight heparin; mAb = monoclonal antibody; PAH = pulmonary arterial hypertension; PDE5 = phosphodiesterase 5; P-gp = P-glycoprotein; PO = oral

Drug-Drug Interaction Considerations When Using Extended Courses of Ritonavir-Boosted Nirmatrelvir (Paxlovid)

The guidance in this document is based on the drug-drug interaction potential of the FDA-approved, 5-day course of ritonavir-boosted nirmatrelvir. Longer treatment courses may be utilized in certain cases (see Special Considerations in People Who Are Immunocompromised). Clinicians should be aware that the drug-drug interaction potential of ritonavir may change based on the duration of treatment. Clinicians should also be aware that:

- Induction properties[6] may become clinically relevant when ritonavir is used for longer durations (i.e., ≥10 days) or chronically (e.g., in people who take HIV protease inhibitors).[7] For example, induction of CYP2C9 and CYP2C19 may decrease warfarin and voriconazole concentrations, and induction of glucuronidation may decrease lamotrigine or valproic acid concentrations.
- The management strategies listed in Box 2 are based on the drug-drug interaction potential of a 5-day treatment course of ritonavir-boosted nirmatrelvir. These strategies may need to be modified when using extended courses. For example, a clinician may need to withhold or reduce the dose of a corticosteroid instead of continuing it as suggested in Box 2. Clinicians may need to adjust monitoring plans for adverse effects or therapeutic drug monitoring in certain patients (e.g., in those receiving tacrolimus). In other cases, the potential risks of withholding certain agents (e.g., chemotherapeutic agents or statins in high-risk individuals) for extended periods to allow for safe coadministration of ritonavir-boosted nirmatrelvir may outweigh the potential benefits of treatment.
- After longer courses of ritonavir-boosted nirmatrelvir are discontinued, drug-drug interactions caused by CYP3A4 inhibition are expected to resolve within 2 to 3 days.[2] Drug-drug interactions caused by induction (e.g., CYP2C9, CYP2C19, UGT) resolve gradually and variably.[8,9]

Clinicians should consult with experts (e.g., pharmacists and physicians with HIV expertise) when using extended courses of ritonavir-boosted nirmatrelvir. The Liverpool COVID-19 Drug Interactions website also provides guidance for managing drug-drug interactions during extended courses (i.e., ≥10 days) of ritonavir-boosted nirmatrelvir.

References

1. Katzenmaier S, Markert C, Riedel KD, et al. Determining the time course of CYP3A inhibition by potent reversible and irreversible CYP3A inhibitors using a limited sampling strategy. *Clin Pharmacol Ther*. 2011;90(5):666-673. Available at: https://www.ncbi.nlm.nih.gov/pubmed/21937987.
2. Stader F, Khoo S, Stoeckle M, et al. Stopping lopinavir/ritonavir in COVID-19 patients: duration of the drug interacting effect. *J Antimicrob Chemother*. 2020;75(10):3084-3086. Available at: https://www.ncbi.nlm.nih.gov/pubmed/32556272.
3. Food and Drug Administration Center for Drug Evaluation and Research. Antimicrobial drugs advisory committee meeting. 2023. Available at: https://www.fda.gov/media/168508/download.
4. Food and Drug Administration. FDA requiring Boxed Warning updated to improve safe use of benzodiazepine drug class. 2020. Available at: https://www.fda.gov/media/142368/download.
5. Li M, Zhu L, Chen L, Li N, Qi F. Assessment of drug-drug interactions between voriconazole and glucocorticoids. *J Chemother*. 2018;30(5):296-303. Available at: https://www.ncbi.nlm.nih.gov/pubmed/30843777.
6. Foisy MM, Yakiwchuk EM, Hughes CA. Induction effects on ritonavir: implications for drug interactions. *Ann Pharmacother*. 2008;42(7):1048-1059. Available at: https://pubmed.ncbi.nlm.nih.gov/18577765.
7. University of Liverpool. Evaluating the interaction risk of COVID-19 therapies. 2022. Available at: https://covid19-druginteractions.org/prescribing_resources. Accessed February 22, 2024.
8. Ramsden D, Fung C, Hariparsad N, et al. Perspectives from the innovation and quality consortium induction working group on factors impacting clinical drug-drug interactions resulting from induction: focus on cytochrome 3A substrates. *Drug Metab Dispos*. 2019;47(10):1206-1221. Available at: https://pubmed.ncbi.nlm.nih.gov/31439574.
9. Marzolini C, Kuritzkes DR, Marra F, et al. Recommendations for the management of drug-drug interactions between the COVID-19 antiviral nirmatrelvir/ritonavir (Paxlovid) and comedications. *Clin Pharmacol Ther*. 2022;112(6):1191-1200. Available at: https://pubmed.ncbi.nlm.nih.gov/35567754.

Management of Nirmatrelvir/Ritonavir (Paxlovid™) Drug-Drug Interactions in Oncology

Paxlovid™ oral tablets consist of a combination of nirmatrelvir and ritonavir co-packaged for use in the treatment of COVID-19. Ritonavir is a protease inhibitor but is not active against SARS-CoV-2, and is administered as a "boosting agent" to slow the metabolism of nirmatrelvir. Ritonavir inhibits the CYP3A-mediated metabolism of nirmatrelvir, resulting in increased plasma concentrations of nirmatrelvir.

The use of ritonavir presents challenges with respect to drug-drug interactions which can potentially impact the efficacy of nirmatrelvir/ritonavir, as well as the safety of coadministered medications. Firstly, nirmatrelvir and ritonavir are substrates of CYP3A4 and are contraindicated in patients taking CYP3A-inducers (current or recent use in last 14-days) since this may significantly decreased concentrations of nirmatrelvir/ritonavir and potential treatment failure. Secondly, ritonavir acts as a potent inhibitor of CYP3A4, P-gp and other CYP isoenzymes and transporters, which may lead to supratherapeutic concentrations for medications that are highly dependent on CYP3A4-mediated metabolism and potentially serious or life-threatening reactions.

A summary of potential drug-drug interactions for medications used in oncology can be found in the table on the proceeding page. There may be some slight variance from the product monograph based upon pharmacokinetic drug principles, the specific dose and duration of nirmatrelvir/ritonavir therapy, and characteristics of individual medications. In oncology, it is common practice to hold certain medications such as cytotoxic chemotherapy, some tyrosine kinase inhibitors (TKIs), cyclin-dependent kinase (CDK) inhibitors, and poly (ADP-ribose) polymerase (PARP) inhibitors during acute infections – including COVID-19. Dose adjustment of many chemotherapeutic agents is problematic due to the need for obtaining a new prescription with temporary new dosing instructions, arranging delivery of specialized chemotherapy agents and then resuming therapy at regular doses. For these medications, the preferred course of action is to hold therapy during treatment with nirmatrelvir/ritonavir. Consultation with an oncology prescriber/pharmacist is therefore recommended and decisions to hold or dose-adjust should be made in conjunction with the patient's oncology team.

The table is not all-inclusive nor all-comprehensive, focussing on the most clinically significant interactions. Therefore, prescribers and pharmacists are encouraged to consult the product monograph, and other resources, such as the guidance document *Nirmatrelvir/Ritonavir: What Prescribers and Pharmacists Need to Know* and the University of Liverpool COVID-19 Drug Interactions Checker.

Drug	Recommendation	Comments
Abemaciclib (Verzenio)	Hold and restart 2 days after completing nirmatrelvir/ritonavir. Alternatively, for patients who have not previously had dose reduction for toxicity, consider a dose reduction to 50 mg once daily with close monitoring for toxicity.	Cyclin-dependent kinase inhibitors are generally held for acute infection. Abemaciclib AUC increased over 3-fold when coadministered with clarithromycin.
Abiraterone	Drug interaction not likely to be clinically relevant. Continue with standard dosing.	In a clinical drug interaction study, ketoconazole 400 mg x 6 days had no impact on abiraterone PK on day 4. Abiraterone was well tolerated.
Acalabrutinib	Hold acalabrutinib and start nirmatrelvir/ritonavir 24 hours after the last acalabrutinib dose. Restart acalabrutinib 2 days after completing nirmatrelvir/ritonavir.	Acalabrutinib AUC increased 5-fold when coadministered with itraconazole.
Afatinib	Drug interaction not likely to be clinically relevant if afatinib and the first daily dose of nirmatrelvir/ritonavir are administered simultaneously. Continue with standard dosing and monitor for toxicity.	Afatinib AUC increased 1.5-fold when administered 1 hour after ritonavir. When ritonavir and afatinib are administered simultaneously or when afatinib is administered 6 hours prior to ritonavir, there was no significant impact on AUC. *Wind et al. Pharmacokinetic drug interactions of afatinib with rifampicin and ritonavir. Clin Drug Investig. 2014;34(3):173-82.*
Alectinib	Drug interaction not likely to be clinically relevant. Continue with standard dosing.	Alectinib and its active metabolite (M4) are metabolized primarily by CYP3A4. Co-administration of strong CYP3A4 inhibitor is anticipated to increase alectinib exposure and decrease M4 exposure. In pharmacokinetic studies with posaconazole, a strong CYP3A4 inhibitor, combined exposure of alectinib and M4 was impacted to a minor extent.
Alpelisib	Drug interaction not likely to be clinically relevant. Continue with standard dosing.	Alpelisib AUC was predicted to increase almost 1.2-fold with administration of ritonavir 100 mg bid using a physiologically-based PK model. *European Medicine Agency Assessment Report: Piqray®.* https://www.ema.europa.eu/en/documents/assessment-report/piqray-epar-public-assessment-report_en.pdf
Anastrozole	Drug interaction not likely to be clinically relevant. Continue with standard dosing.	Metabolism to hydroxyanastrozole primarily via CYP3A4 and glucuronidation to anastrozole N-glucuronide by UGT1A4. These metabolites are pharmacologically inactive. Potential for increased anastrozole concentrations via CYP3A4 inhibition by ritonavir. Clinical significance unknown.
Apalutamide (Erleada)	Contraindicated (use within past 14 days).<br>Use alternative COVID agent.<br>Do not use nirmatrelvir/ritonavir.	Coadministration has not been studied. Apalutamide is a strong inducer of CYP3A4 (92% decrease in AUC of midazolam).

Drug	Recommendation	Comments
Avapritinib	Contraindicated.<br>Use alternative COVID agent.<br>Do not use nirmatrelvir/ritonavir.	Predicted 6-fold increase in avapritinib AUC when co-administered with itraconazole. Given long avapritinib half-life (32-57 hours), interaction is unlikely to be mitigated by holding avapritinib.
Axitinib	Hold and restart 2 days after completing nirmatrelvir/ritonavir. Alternatively, consider reducing dose by 50% if used concomitantly and monitor for toxicity. Resume previous dose 2 days after completing nirmatrelvir/ritonavir.	Axitinib AUC increased 2-fold when coadministered with ketoconazole.
Azacitidine	No interaction expected.	Azacitidine has no known induction or inhibition effects on cytochrome P450 enzymes and does not undergo P450 mediated metabolism.
Bevacizumab	No interaction expected.	Coadministration has not been studied. A clinically significant pharmacokinetic interaction is unlikely as bevacizumab is metabolized via proteolytic catabolism.
Bexarotene	Contraindicated (use within past 14 days).<br>Use alternative COVID agent.<br>Do not use nirmatrelvir/ritonavir.	Coadministration has not been studied. Bexarotene is a moderate CYP3A4 inducer and may decrease exposure to nirmatrelvir/ritonavir.
Bicalutamide	Drug interaction not likely to be clinically relevant. Continue with standard dosing.	A number of clinical studies show the magnitude of any inhibition is unlikely to be of clinical significance for the majority of substances metabolised by CYP P450.
Binimetinib	Drug interaction not likely to be clinically relevant. Continue with standard dosing.	Coadministration has not been studied. A clinically significant PK interaction is not expected with short duration of co-administration as binimetinib is primarily metabolized by UGT1A1.
Bortezomib	Hold and restart 2 days after completing nirmatrelvir/ritonavir. Alternatively, may consider co-administration with close monitoring for bortezomib related toxicity.	Bortezomib is generally held during acute infection. Bortezomib AUC increased almost 1.4-fold when coadministered with ketoconazole.
Bosutinib (Bosulif)	Hold bosutinib and start nirmatrelvir/ritonavir 24 hours after the last bosutinib dose. Restart bosutinib 2 days after completing nirmatrelvir/ritonavir.	Bosutinib AUC increased almost 9-fold when coadministered with ketoconazole.
Brigatinib	Hold and restart 2 days after completing nirmatrelvir/ritonavir. Alternatively, consider reducing dose by 50% if used concomitantly and monitor for toxicity. Resume previous dose 2 days after completing nirmatrelvir/ritonavir.	Brigatinib AUC increased 2-fold when coadministered with itraconazole.

Drug	Recommendation	Comments
Cabozantinib	Hold and restart 2 days after completing nirmatrelvir/ritonavir. Alternatively, consider reducing dose by 20 mg if used concomitantly and monitor for toxicity. Resume previous dose 2 days after completing nirmatrelvir/ritonavir.	Cabozantinib AUC increased almost 1.4-fold when coadministered in the presence of steady-state ketoconazole 400mg daily.
Capecitabine	No interaction expected.	Coadministration has not been studied but based on metabolism and clearance, a clinically significant interaction is not expected.
Capmatinib	Drug interaction not likely to be clinically relevant. Continue with standard dosing.	Capmatinib AUC increased 1.4-fold when coadministered with itraconazole. No effect on the capmatinib Cmax was noted.
Cedazuridine/ Decitabine	No interaction expected.	Coadministration has not been studied but based on metabolism and clearance a clinically significant interaction is not expected.
Ceritinib (Zykadia)	Hold and restart 2 days after completing nirmatrelvir/ritonavir. Alternatively, consider reducing ceritinib dose by 33% if used concomitantly and monitor for toxicity.	Canadian monograph recommends to avoid concomitant use. However, US monograph suggests reducing dose by 33%, rounded to nearest 150 mg dosage strength.<br>Zykadia (U.S.) Prescribing Information. Accessed February 8, 2022.<br>https://www.accessdata.fda.gov/drugsatfda_docs/label/2019/205755s016lbl.pdf<br>Decision to hold or dose-adjust ceritinib should be made in conjunction with the patient's oncologist.<br>Ceritinib AUC increased 3-fold when single dose coadministered with ketoconazole.
Chlorambucil	No interaction expected.	Coadministration has not been studied and little is known about chlorambucil metabolism in humans. Based on available data, a clinically significant interaction is not expected.
Cobimetinib (Cotellic)	Hold cobimetinib and start nirmatrelvir/ritonavir 24 hours after the last cobimetinib dose. Restart cobimetinib 2 days after completing nirmatrelvir/ritonavir.	Cobimetinib AUC increased almost 7-fold when coadministered with ketoconazole.
Crizotinib	Hold and restart 2 days after completing nirmatrelvir/ritonavir. Alternatively, consider reducing dose by at least 50% if used concomitantly and monitor for toxicity. Resume previous dose 2 days after completing nirmatrelvir/ritonavir.	Crizotinib AUC increased 3.2-fold when coadministered with ketoconazole.

Drug	Recommendation	Comments
Cyclophosphamide	Drug interaction not likely to be clinically relevant. Continue with standard dosing.	Based on metabolism and clearance, clinically relevant interaction is not expected with short course of nirmatrelvir/ritonavir. Activation of cyclophosphamide (major pathway) to 4-hydroxycyclophosphamide is catalyzed by CYPs 2B6 (major), 2C9 and 3A4. Inactivation (minor, 10%) to the neurotoxic metabolite is performed mainly by CYP3A4.
Cytarabine	No interaction expected.	Coadministration has not been studied, but no PK interaction is expected as cytarabine is primarily metabolized via cytidine deaminase.
Dabrafenib (Tafinlar)	Contraindicated (use within past 14 days).<br>Use alternative COVID agent.<br>Do not use nirmatrelvir/ritonavir.	Dabrafenib is a moderate to strong in vivo inducer of CYP3A4. Coadministration may decrease exposure to nirmatrelvir/ritonavir.
Dacomitinib	No interaction expected.	Coadministration has not been studied. A clinically significant pharmacokinetic interaction is unlikely based on metabolism and clearance.
Darolutamide	Hold and restart 2 days after completing nirmatrelvir/ritonavir. Alternatively, monitor for daralutamide related toxicity and interrupt therapy or consider dose reduction if necessary.	Darolutamide AUC increased 1.7-fold when coadministered with itraconazole. Given darolutamide is relatively well tolerated, empiric dose adjustment is not suggested.
Dasatinib (Sprycel)	Chronic phase chronic myelogenous leukemia (CML):<br>Hold and restart 2 days after completing nirmatrelvir/ritonavir. Alternatively, consider reducing dasatinib dose to 20 to 40 mg if used concomitantly and monitor for toxicity.<br>Accelerated or blast phase CML:<br>Do not coadminister; use alternate COVID-19 therapy.	Dasatinib AUC increased 5-fold when coadministered with ketoconazole.
Degarelix	No interaction expected.	Coadministration has not been studied. A clinically significant pharmacokinetic interaction is unlikely as degarelix is metabolized via peptide hydrolysis.

Drug	Recommendation	Comments
Dexamethasone	High dose (≥20 mg daily): Reduce dexamethasone dose by 50% and resume usual dose 2 days after completing nirmatrelvir/ritonavir.<br>Low dose (<20 mg daily): Continue with usual dose during nirmatrelvir/ritonavir.	Dexamethasone AUC increased almost 3-fold when coadministered with voriconazole.<br>Li M, Zhu L, Chen L et al. Assessment of drug-drug interactions between voriconazole and glucocorticoids. J Chemother. 2018;30(5):296-303. doi: 10.1080/1120009X.2018.1506693.<br>Potential for risk of dexamethasone toxicity with high doses (≥20 mg daily).<br>Clinically significant interaction is not expected with dexamethasone at low doses, including when used for COVID-19 treatment.
Duvelisib	Hold and restart 2 days after completing nirmatrelvir/ritonavir. Alternatively, consider reducing dose to 15 mg BID if used concomitantly and monitor for toxicity. Resume previous dose 2 days after completing nirmatrelvir/ritonavir.<br>Note: if patient is already receiving 15 mg BID due to previous reduction for toxicity, do not coadminister and consider alternate COVID-19 therapy if unable to hold.	Depending on disease volume, abrupt discontinuation of duvelisib may result in disease flare in patients with CLL.<br>Based on physiologically-based PK modeling and simulation, the increase in exposure to duvelisib is estimated to be almost 2-fold at steady state when concomitantly used with strong CYP3A4 inhibitors.
Enasidenib	Contraindicated (use within past 14 days).<br>Use alternative COVID agent.<br>Do not use nirmatrelvir/ritonavir.	Enasidenib induces CYP3A4. The clinical significance of this potential drug interaction is unknown at this time. Given lack of available data regarding magnitude of induction potential, suggest not to coadminister due to potential to decrease in nirmatrelvir/ritonavir.
Encorafenib (Braftovi)	Hold encorafenib and restart 2 days after completing nirmatrelvir/ritonavir.<br>Alternatively, consider reducing encorafenib dose as follows and monitoring for toxicity:<br>If taking 450 mg per day: reduce to 150 mg daily.<br>If taking 150 to 300 mg per day: reduce dose to 75 mg daily.<br>Resume usual encorafenib dose 2 days after completing nirmatrelvir/ritonavir.	Encorafenib AUC increased 3-fold when coadministered with posaconazole.
Entrectinib	Hold and restart 2 days after completing nirmatrelvir/ritonavir. Alternatively, consider reducing dose to 100 mg daily if used concomitantly and monitor for toxicity. Resume previous dose 2 days after completing nirmatrelvir/ritonavir.	Entrectinib AUC increased 6-fold when coadministered with itraconazole.

Drug	Recommendation	Comments
Enzalutamide (Xtandi)	Contraindicated (use within past 8 weeks).<br>Use alternative COVID agent.<br>Do not use nirmatrelvir/ritonavir.	Enzalutamide is a strong CYP3A4 inducer and may reduce concentrations of nirmatrelvir/ritonavir. Given long terminal half-life of enzalutamide (~6 days), enzyme induction is expected to persist. A physiologically-based pharmacokinetic model predicts that CYP3A4 activity does not return to baseline until at least 8 weeks after enzalutamide discontinuation.<br>*Narayanan et al. Application of a "Fit for Purpose" PBPK Model to Investigate the CYP3A4 Induction Potential of Enzalutamide. Drug Metab Lett. 2016;10(3):172-179.*
Erdafitinib	Hold and restart 2 days after completing nirmatrelvir/ritonavir. Alternatively, monitor for erdafitinib related toxicity if used concomitantly and interrupt therapy or consider dose reduction if necessary.	Erdafitinib AUC increased 1.3-fold when coadministered with itraconazole.
Erlotinib	Hold and restart 2 days after completing nirmatrelvir/ritonavir. Alternatively, consider reducing dose in 50 mg decrements if toxicity occurs. Restart previous dose 2 days after completion of nirmatrelvir/ritonavir.	Coadministration of erlotinib and ketoconazole led to 86% increase in erlotinib AUC.
Etoposide	Hold and restart 2 days after completing nirmatrelvir/ritonavir. Alternatively, monitor for etoposide related toxicity if used concomitantly and interrupt therapy or consider dose reduction if necessary.	Co-administration has not been studied. Etoposide is metabolized by CYP3A4 and undergoes P-gp medicated transport, therefore coadministration with nirmatrelvir/ritonavir theoretically may increase etoposide exposure.
Everolimus	Contraindicated.<br>Use alternative COVID agent.<br>Do not use nirmatrelvir/ritonavir.	Everolimus AUC increased 15-fold when coadministered with ketoconazole. Given long terminal half-life (30 hours), holding everolimus is unlikely to mitigate effects of this interaction.
Exemestane	Drug interaction not likely to be clinically relevant. Continue with standard dosing.	Exemestane is metabolized by CYP3A4, but coadministration with ketoconazole did not result in clinically significant changes in exemestane exposure.
Fedratinib	Hold and restart 2 days after completing nirmatrelvir/ritonavir. Alternatively, consider reducing dose to 200 mg daily depending on indication, tolerability, and previously planned dose and monitor for toxicity. Discontinue fedratinib if the patient is unable to tolerate a dose of 200mg daily.	Fedratinib AUC is predicted to increase by 2.5-fold when coadministered with ritonavir 100 mg BID based on physiologically-based PK modeling simulations.
Fludarabine	No interaction expected.	Coadministration has not been studied. A clinically significant pharmacokinetic interaction is unlikely as fludarabine is metabolized via non-CYP P450 pathways.

Drug	Recommendation	Comments
Flutamide	Hold and restart 2 days after completing nirmatrelvir/ritonavir.	Potential for increased/decreased flutamide concentrations secondary to inhibition of CYP3A4 or induction of CYP1A2 by lopinavir/ritonavir; clinical significance is unknown. Monitor for flutamide efficacy and toxicity. Studies show the principal role of CYP1A2 is in the metabolism of flutamide to 2-hydroxyflutamide (active metabolite).
Fostamatinib (Tavalisse)	No dose-adjustment is required, HOWEVER, monitor for toxicity including diarrhea, hypertension, hepatotoxicity, and neutropenia. If significant toxicity occurs, consider interruption of fostamatinib with reintroduction 2 days after completing nirmatrelvir/ritonavir.	Fostamatinib active metabolite AUC increased 2-fold when administered with ketoconazole.
Fulvestrant	Drug interaction not likely to be clinically relevant. Continue with standard dosing.	Results from a clinical study with ketoconazole (400 mg daily), a potent inhibitor of CYP3A4, indicate that there is no clinically relevant change in the PK of an 8 mg IV dose of fulvestrant. Fulvestrant is a minor substrate of CYP 3A4; dosage adjustments are not considered necessary during co-administration with CYP 3A4 inhibitors or inducers.
Gefitinib	Hold and restart 2 days after completing nirmatrelvir/ritonavir. Alternatively, monitor for gefitinib related toxicity if used concomitantly and interrupt therapy or consider dose reduction if necessary.	In healthy volunteers, gefitinib AUC increased 1.8-fold when coadministered with itraconazole. In a developed physiologically-based PK model, steady state gefitinib concentrations were simulated in a virtual population of 50 individuals receiving gefitinib 250 mg daily with and without darunavir 800 mg/ritonavir 100 mg daily, efavirenz 600 mg daily, or etravirine 200 mg twice daily. Gefitinib AUC was increased 5.5-fold when coadministered with darunavir/ritonavir, and was still increased by 2.79-fold after halving the gefitinib dose to 125 mg daily. However, these concentrations were still considered to be within the therapeutic range. Some references recommend reducing gefitinib to 125mg daily, however, note that gefitinib is a hazardous drug, so handling safety in splitting tablets should be considered.
Gilteritinib	Hold and restart 2 days after completing nirmatrelvir/ritonavir. Alternatively, monitor for gilteritinib related toxicity if used concomitantly and interrupt therapy or consider dose reduction if necessary.	Gilteritinib AUC increased 2.2-fold when coadministered with itraconazole.

Drug	Recommendation	Comments
Glasdegib	Hold and restart 2 days after completing nirmatrelvir/ritonavir. Alternatively, monitor for glasdegib related toxicity if used concomitantly and interrupt therapy or consider dose reduction if necessary.	Glasdegib AUC increased 2.4-fold when coadministered with ketoconazole dosed at 400 mg once daily for 7 days in healthy volunteers.
Goserelin	No interaction expected.	Coadministration has not been studied. A clinically significant pharmacokinetic interaction is unlikely as goserelin is metabolized via non-CYP P450 pathways.
Hydroxyurea	No interaction expected.	Coadministration has not been studied. A clinically significant pharmacokinetic interaction is unlikely as hydroxyurea is metabolized via non-CYP P450 pathways.
Ibrutinib (Imbruvica)	Consider alternate COVID-19 therapy.<br>Alternatively, consider holding ibrutinib and starting nirmatrelvir/ritonavir 12 hours after the last ibrutinib dose. Restart ibrutinib 2 days after completing nirmatrelvir/ritonavir.	Ibrutinib AUC increased 26-fold when coadministered with ketoconazole. It may be dangerous to interrupt therapy in patients with high volume chronic lymphocytic leukemia or mantle cell lymphoma due to disease flare and/or cytokine release.
Idelalisib	Hold and restart 2 days after completing nirmatrelvir/ritonavir. Alternatively, monitor for idelalisib related toxicity if used concomitantly and interrupt therapy or consider dose reduction if necessary.	Idelalisib AUC increased 1.8-fold when coadministered with ketoconazole.
Imatinib	Hold and restart 2 days after completing nirmatrelvir/ritonavir. Alternatively, monitor for imatinib related toxicity if used concomitantly and interrupt therapy or consider dose reduction if necessary.	Imatinib AUC increased 1.4-fold when coadministered with ketoconazole. In another pharmacokinetic study of 11 cancer patients, coadministration with ritonavir (600 mg for 3 days) did not alter imatinib (400 mg to 800 mg daily for at least 2 months) steady-state concentrations, but did increase exposure to the imatinib metabolite (CGP74588) 1.4-fold.
Infigratinib	Contraindicated.<br>Use alternative COVID agent.<br>Do not use nirmatrelvir/ritonavir.	Infigratinib AUC increased 7-fold when coadministered with multiple doses of itraconazole. BHS697 (active metabolite) increased almost 3-fold when coadministered with multiple doses of itraconazole. Given long terminal half-life of parent drug (33 hours), holding infigratinib is unlikely to mitigate effects of this interaction.

Drug	Recommendation	Comments
Isotretinoin	Hold and restart 2 days after completing nirmatrelvir/ritonavir. Alternatively, monitor for isotretinoin related toxicity if used concomitantly and interrupt therapy or consider dose reduction if necessary.	Coadministration has not been studied. Ritonavir could potentially increase isotretinoin concentrations by inhibition of CYP2C8 and CYP3A4. However, plasma retinoid concentrations in one patient treated with isotretinoin were substantially lower after the start of antiviral therapy (indinavir/ ritonavir 800/800 mg daily dose + zidovudine/lamivudine). This was unexpected and the reason is unclear.
Ivosidenib	Contraindicated (use within past 14 days).<br>Use alternative COVID agent.<br>Do not use nirmatrelvir/ritonavir.	Ivosidenib induces CYP3A4 and is itself a substrate of CYP3A4. Simulations suggest that concomitant use of ivosidenib (500 mg once daily) and the sensitive CYP3A substrate midazolam (single 5-mg dose) decreases AUC and peak plasma concentration of midazolam by 83 and 74%, respectively. Given long terminal half-life of ivosidenib (~2-5 days), enzyme induction is expected to persist. Ivosidenib AUC increased 2.7-fold when coadministered with itraconazole.
Ixazomib	No interaction expected	Coadministration with strong CYP3A4 and CYP1A2 inhibitors will likely not result in clinically significant interactions.
Lanreotide	Drug interaction not likely to be clinically relevant. Continue with standard dosing.	Product monograph mentions somatostatin analogues might decrease metabolic clearance of compounds metabolized by CYP 450 enzymes, which might be due to suppression of growth hormone. Paxlovid is a CYP 3A4 substrate.
Lapatinib	Hold and restart 2 days after completing nirmatrelvir/ritonavir. Alternatively, consider reducing dose lapatinib dose from 1250mg to 500mg daily if used concomitantly and monitor for toxicity. A 7-day washout period is recommended before the lapatinib dose is readjusted upwards.	Lapatinib AUC increased 3.6-fold when coadministered with ketoconazole.
Larotrectinib	Hold and restart 2 days after completing nirmatrelvir/ritonavir. Alternatively, consider reducing larotrictinib dose by 50% if used concomitantly and monitor for toxicity. Restart previous dose 2 days after completion of nirmatrelvir/ritonavir.	Larotrectinib AUC increased 4.3-fold when coadministered with itraconazole.
Lenalidomide (Revlimid)	No interaction expected.	Lenalidomide is not a substrate, inhibitor or inducer of cytochrome P450 enzymes. Hence, coadministration of cytochrome P450 substrates or inhibitors with lenalidomide is not likely to result in clinically relevant drug-drug interactions.

Drug	Recommendation	Comments
Lenvatinib	No interaction expected.	Lenvatinib may be co-administered without dose adjustment with CYP3A inhibitors and CYP3A inducers. Avoid coadministration of lenvatinib with other drugs known to prolong the QT interval because of risk for additive effects on the QT interval that can lead to serious cardiac adverse effects, including torsade de pointes.
Letrozole (Femara)	Drug interaction not likely to be clinically relevant. Continue with standard dosing. Monitor for letrozole related toxicity if used concomitantly and interrupt therapy or consider dose reduction if necessary.	Coadministration has not been studied. Letrozole is metabolised by CYP3A4 and CYP2A6 to carbinol, an inactive metabolite. Coadministration could potentially increase letrozole concentrations and thus increase the occurrence of side effects. A clinical interaction study with cimetidine (a non-specific inhibitor of CYP2C19 and CYP3A4) indicated that coadministration with letrozole does not result in a clinically significant drug interaction
Leuprolide	No interaction expected.	Coadministration has not been studied. A clinically significant pharmacokinetic interaction is unlikely as leuprolide is metabolized via peptide hydrolysis.
Lomustine	Drug interaction not likely to be clinically relevant. Continue with standard dosing. Monitor for lomustine related toxicity if used concomitantly and interrupt therapy or consider dose reduction if necessary.	Coadministration has not been studied. Potential for increased lomustine exposures with CYP2D6 inhibitors; ritonavir is a weak inhibitor of CYP2D6. Clinical significance unknown.
Lorlatinib (Lorbrena)	Contraindicated (use within past 14 days).<br>Use alternative COVID agent.<br>Do not use nirmatrelvir/ritonavir.	Lorlatinib induces CYP3A4 and is itself a substrate of CYP3A4. Lorlatinib 150 mg orally once daily for 15 days decreased the AUC by 64% of a single oral 2 mg dose of midazolam. Given long terminal half-life of lorlatinib (~24 hours), enzyme induction is expected to persist.
Melphalan	No interaction expected.	Coadministration has not been studied. A clinically significant pharmacokinetic interaction is unlikely as melphalan is metabolized via chemical hydrolysis.
Mercaptopurine	No interaction expected.	Coadministration has not been studied. A clinically significant pharmacokinetic interaction is unlikely as mercaptopurine is metabolized via non-CYP P450 pathways.
Methotrexate	No interaction expected. Monitor for methotrexate related toxicity if used concomitantly and interrupt therapy or consider dose reduction if necessary.	Coadministration has not been studied. A clinically significant pharmacokinetic interaction is unlikely as methotrexate is metabolized via non-CYP P450 pathways.

Drug	Recommendation	Comments
Midostaurin	Hold and restart 2 days after completing nirmatrelvir/ritonavir. Alternatively, consider reducing dose by 50% of ideal dose if used concomitantly and monitor for toxicity. Restart previous dose 2 days after completion of nirmatrelvir/ritonavir.	Midostaurin AUC increased 10.4-fold when coadministered with ketoconazole. Monitor closely for increased toxicities especially during the first week of consecutive midostaurin administration in the advanced systemic mastocytosis population, and during first week of midostaurin administration in each cycle of chemotherapy in the AML population.
Mitotane (Lysodren)	Contraindicated (use within past 3-6 months).<br>Use alternative COVID agent.<br>Do not use nirmatrelvir/ritonavir.	Coadministration has not been studied. Mitotane is a strong inducer of CYP3A4. Given long terminal half-life of mitotane (18 to 159 days), enzyme induction is expected to persist.
Mobocertinib	Hold and restart 2 days after completing nirmatrelvir/ritonavir.	Mobocertinib AUC increased 6.3-fold when coadministered with itraconazole.
Neratinib (Nerlynx)	Hold neratinib and start nirmatrelvir/ritonavir 24 hours after the last neratinib dose. Restart neratinib 2 days after completing nirmatrelvir/ritonavir.	Neratinib AUC increased 4.8-fold when administered with ketoconazole.
Nilotinib (Tasigna)	Chronic phase chronic myelogenous leukemia (CML):<br>Hold nilotinib if possible, and restart 2 days after completing nirmatrelvir/ritonavir. Alternatively, consider dose reduction to 400 mg PO daily and monitor for toxicity.<br>Accelerated or blast phase CML:<br>Do not coadminister, consider alternate COVID-19 therapy.	Nilotinib AUC increased 3-fold when administered with ketoconazole.
Nilutamide	No interaction expected.	Coadministration has not been studied. A clinically significant pharmacokinetic interaction is unlikely as nilutamide is metabolized via non-CYP P450 pathways.
Niraparib	No interaction expected.	Coadministration has not been studied. A clinically significant pharmacokinetic interaction is unlikely as niraparib is metabolized via non-CYP P450 pathways.
Octreotide	Drug interaction not likely to be clinically relevant. Continue with standard dosing.	Somatostatin analogues may decrease the clearance of compounds metabolized by CYP3A4 through the suppression of growth hormone. Concurrent use of octreotide with substrates of CYP3A4 should be done cautiously, particularly if the substrate drug has a low therapeutic index.

Drug	Recommendation	Comments
Olaparib	Hold and restart 2 days after completing nirmatrelvir/ritonavir. Alternatively, consider reducing olaparib tablet dose to 100 mg twice daily, or reducing olaparib capsule dose to 150 mg twice daily. Monitor for olaparib related toxicity if used concomitantly and interrupt therapy if necessary. Restart previous dose 2 days after completion of nirmatrelvir/ritonavir.	Olaparib AUC increased 2.7-fold when coadministered with itraconazole.
Osimertinib	Drug interaction not likely to be clinically relevant. Continue with standard dosing.	Osimertinib AUC increased 1.2-fold when coadministered with itraconazole. Given the inter-patient variability of 46% in the osimertinib exposure in the population PK analysis, this change of 24% is not clinically significant. Due to the dose proportional, linear and time independent PK of osimertinib, the effect of a strong CYP3A4 inhibitor at steady state is likely to be similar to that seen after a single dose. Hence, CYP3A4 inhibitors are unlikely to affect the exposure of Osimertinib.
Palbociclib	Hold and restart 2 days after completing nirmatrelvir/ritonavir. Alternatively, consider reducing palbociclib dose to 75 mg daily and monitor for toxicity. Restart previous dose 2 days after completion of nirmatrelvir/ritonavir.	Palbociclib AUC increased almost 1.9-fold when coadministered with itraconazole.
Panobinostat	Hold and restart 2 days after completing nirmatrelvir/ritonavir. Alternatively, consider reducing to 10 mg or interrupt therapy as necessary and monitor for toxicity. Restart previous dose 2 days after completion of nirmatrelvir/ritonavir.	Panobinostat AUC increased almost 1.8-fold when coadministered with ketoconazole.
Pazopanib	Hold and restart 2 days after completing nirmatrelvir/ritonavir. Alternatively, consider reducing dose to 400 mg or interrupt therapy as necessary and monitor for toxicity. Reduce dose further if necessary. Do not use doses higher than 400mg. Restart previous dose 2 days after completion of nirmatrelvir/ritonavir.	Pazopanib AUC increased 1.7-fold when coadministered with ketoconazole. More adverse events were observed when pazopanib was administered in combination with ketoconazole than when pazopanib was administered alone, which included cases of severe hypertension. In the presence of a strong CYP3A4 and P-gp inhibitor, a dose reduction to 400 mg daily may result in systemic exposure higher than that observed after administration of 800 mg pazopanib daily alone. In a minority (25%) of patients the dose of 400 mg pazopanib daily in the presence of ketoconazole resulted in systemic exposure greater than the highest systemic exposure observed after administration of 800 mg pazopanib daily alone.

Drug	Recommendation	Comments
Pemigatinib	Hold and restart 2 days after completing nirmatrelvir/ritonavir. Alternatively, consider reducing dose as follows:<br>If taking 13.5 mg daily for the first 14 days of each cycle, decrease the pemigatinib dose to 9 mg;<br>If taking 9 mg daily for the first 14 days of each cycle, decrease the pemigatinib dose to 4.5 mg.<br>Monitor for pemigatinib related toxicity if used concomitantly and interrupt therapy if necessary. Restart previous dose 2 days after completion of nirmatrelvir/ritonavir.	Pemigatinib AUC increased almost 1.9-fold when coadministered with itraconazole.
Pexidartinib	Contraindicated (use within past 14 days).<br>Use alternative COVID agent.<br>Do not use nirmatrelvir/ritonavir.	Coadministration has not been studied. Pexidartinib is a moderate CYP3A4 inducer and may decrease exposure to nirmatrelvir/ritonavir. Additionally, pexidartinib AUC increased 1.7-fold when coadministered with itraconazole.
Pomalidomide	Drug interaction not likely to be clinically relevant. Continue with standard dosing.	Pomalidomide AUC increased 1.2-fold when coadministered with ketoconazole.
Ponatinib	Hold and restart 2 days after completing nirmatrelvir/ritonavir. Alternatively, consider reducing dose as follows:<br>If taking 45 mg daily, reduce to 30 mg daily;<br>If taking 30 mg daily, reduce to 15 mg daily;<br>If taking 15 mg daily, reduce to 10 mg daily.<br>If taking 10 mg daily, do not use nirmatrelvir/ritonavir. avoid concomitant use with strong CYP3A4 inhibitors. Monitor for ponatinib related toxicity if used concomitantly and interrupt therapy if necessary. Restart previous dose 2 days after completion of nirmatrelvir/ritonavir.	Ponatinib AUC increased 1.8-fold when coadministered with ketoconazole.
Pralsetinib	Hold and restart 2 days after completing nirmatrelvir/ritonavir. Alternatively, consider reducing dose as follows:<br>If taking 400 mg or 300 mg daily, reduce to 200 mg daily;<br>If taking 200 mg daily, reduce to 100 mg once daily.<br>Monitor for pralsetinib related toxicity if used concomitantly and interrupt therapy if necessary. Restart previous dose 2 days after completion of nirmatrelvir/ritonavir.	Pralsetinib AUC increased 3.5-fold when coadministered with itraconazole.
Procarbazine	Drug interaction not likely to be clinically relevant. Continue with standard dosing.	Coadministration has not been studied. Procarbazine is metabolized to azoprocarbazine by CYP450 (mainly CYP2B and 1A) and monoamine oxidase. Ritonavir could potentially decrease procarbazine concentrations due to induction of CYPs 2B and 1A.

Drug	Recommendation	Comments
Regorafenib	Contraindicated.<br>Use alternative COVID agent.<br>Do not use nirmatrelvir/ritonavir.	Regorafenib AUC increased 1.3-fold when coadministered with ketoconazole. However, the AUC of both the active M-2 (N-oxide) and M-5 (N-oxide and N-desmethyl) metabolites decreased by 93%.
Relugolix	Hold and restart 2 days after completing nirmatrelvir/ritonavir. If coadministration is unavoidable, take relugolix at least 6 hours prior to the P-gp inhibitor and monitor patients more frequently for adverse reactions. Treatment with relugolix may be interrupted for up to two weeks if a short course of treatment with a P-gp inhibitor is required. Monitor for relugolix related toxicity if used concomitantly and interrupt therapy if necessary. Restart previous dose 2 days after completion of nirmatrelvir/ritonavir. However, if treatment with relugolix is interrupted for more than 7 days, resume administration of relugolix with a 360 mg loading dose on the first day, followed by 120 mg once daily.	Relugolix AUC increased 6.2-fold when coadministered with erythromycin (P-gp and moderate CYP3A inhibitor).
Ribociclib	Hold and restart 2 days after completing nirmatrelvir/ritonavir. Alternatively, consider reducing dose to 200 mg daily; however, there are no clinical data with this dose adjustment. Monitor for ribociclib related toxicity if used concomitantly and interrupt therapy if necessary. Restart previous dose 2 days after completion of nirmatrelvir/ritonavir.	Ribociclib AUC increased 3.2-fold when coadministered with ritonavir.
Ripretinib	Hold and restart 2 days after completing nirmatrelvir/ritonavir. Alternatively, monitor for ripretinib related toxicity if used concomitantly and interrupt therapy or consider dose reduction if necessary.	Ripretinib AUC increased 2-fold when coadministered with itraconazole. The AUC of the active metabolite, DP-5439, also increased 2-fold in the presence of itraconazole.
Rucaparib	Hold and restart 2 days after completing nirmatrelvir/ritonavir. Alternatively, monitor for rucaparib related toxicity if used concomitantly and interrupt therapy or consider dose reduction if necessary.	Coadministration has not been studied. Potential for increased rucaparib exposures with CYP2D6 inhibitors; ritonavir is a weak inhibitor of CYP2D6. In vitro, rucaparib is metabolized primarily by CYP2D6 and to a lesser extent by CYP1A2 and CYP3A4. Clinical significance unknown.
Ruxolitinib	Hold and restart 2 days after completing nirmatrelvir/ritonavir. Alternatively, consider reducing dose by 50% if used concomitantly and monitor for toxicity. Resume previous dose 2 days after completing nirmatrelvir/ritonavir.	Ruxolitinib AUC increased 1.9-fold when coadministered with ketoconazole.

Drug	Recommendation	Comments
Selinexor	Hold and start nirmatrelvir/ritonavir 24 hours after the last selinexor dose. Restart selinexor 2 days after completing nirmatrelvir/ritonavir.	Coadministration has not been studied. Selinexor is metabolized by CYP3A4, multiple UDP-glucuronosyltransferases (UGTs) and glutathione S-transferases (GSTs). Selinexor is not a CYP3A4, CYP1A2, or CYP2B6 inducer. In vivo implications are not known.
Selpercatinib	Hold and restart 2 days after completing nirmatrelvir/ritonavir Alternatively, consider reducing dose from 120 mg twice daily to 40 mg twice daily, or from 160 mg twice daily to 80 mg twice daily. Monitor for selpercatinib related toxicity if used concomitantly and interrupt therapy if necessary. Restart previous dose 2 days after completion of nirmatrelvir/ritonavir.	Selpercatinib AUC increased 2.3-fold when coadministered with itraconazole.
Sonidegib	Contraindicated.<br>Use alternative COVID agent.<br>Do not use nirmatrelvir/ritonavir.	Sonidegib AUC increased 2.2-fold when coadministered with ketoconazole. The magnitude of exposure change was estimated to be even higher following repeated doses of sonidegib and with continuous dosing of ketoconazole. Given long half-life (28 days), holding sonidegib is unlikely to mitigate effects of this interaction.
Sorafenib	Contraindicated.<br>Use alternative COVID agent.<br>Do not use nirmatrelvir/ritonavir.	Sorafenib is metabolized to a moderate extent by CYP3A4 and UGT1A9, UGT1A1. Sorafenib AUC was minimally changed when coadministered with ketoconazole. However, concentrations of the active N-oxide metabolite (which accounts for only 9 to 16% of circulating metabolites and is thought to be generated by CYP3A4), were substantially decreased by ketoconazole. In another small study of 10 patients with HIV and Kaposi sarcoma, when sorafenib 200mg daily was coadministered with ritonavir, the sorafenib AUC was 28% lower compared with the sorafenib AUC in 2 patients who received sorafenib 200 mg twice daily alone. Concentrations of the active N-oxide metabolite were 74% lower in the ritonavir group. The study had to be terminated early due to poor tolerance, which could possibly be related to inhibition of CYP3A4 by ritonavir leading to the formation of more toxic metabolites.
Sotorasib	Contraindicated (use within past 14 days).<br>Use alternative COVID agent.<br>Do not use nirmatrelvir/ritonavir.	Sotorasib is a moderate CYP3A4 inducer and may reduce concentrations of nirmatrelvir/ritonavir.

Drug	Recommendation	Comments
Sunitinib	Hold and restart 2 days after completing nirmatrelvir/ritonavir. Alternatively, consider reducing sunitinib dose to a minimum of 37.5mg daily for 4 weeks on treatment, then 2 weeks off when treating gastrointestinal stromal tumor (GIST) or renal cell carcinoma (RCC). Decrease the sunitinib dose to a minimum of 25mg daily when treating pancreatic neuroendocrine tumor (PNET). Sunitinib dose may be reduced in 12.5 mg per day increments down to 25 mg per day in patients receiving CYP3A4 inhibitors. Monitor for sunitinib related toxicity if used concomitantly and interrupt therapy if necessary. Restart previous dose 2 days after completion of nirmatrelvir/ritonavir.	Sunitinib and its primary active metabolite's AUC increased 1.5-fold when coadministered with ketoconazole.
Talazoparib	Hold and restart 2 days after completing nirmatrelvir/ritonavir. Alternatively, if coadministration with a strong P-gp inhibitor is unavoidable, the dose should be reduced to the next lower dose. Monitor for talazoparib related toxicity if used concomitantly and interrupt therapy if necessary. Restart previous dose 2 days after completion of nirmatrelvir/ritonavir.	Talazoparib AUC increased 1.5-fold when coadministered with itraconazole. Talazoparib is a major substrate of P-gp.
Tamoxifen	Drug interaction not likely to be clinically relevant. Continue with standard dosing.	Some data suggest that tamoxifen may induce CYP metabolizing enzymes, although the clinical impact of this induction effect is not well established. Serum concentrations of letrozole (a CYP3A4 and 2D6 substrate) were decreased 38% in 12 female patients following 6 weeks of concomitant administration of tamoxifen. Additionally, ritonavir may increase tamoxifen parent concentration due to inhibition of CYP3A4 and decrease of the active metabolites (4-hydroxytamoxifen and endoxifen) due to potential moderate inhibition of CYP2D6 by ritonavir. The clinical significance of these interactions with short duration of use is expected to be low.
Temozolomide	No interaction expected.	Clearance of temozolomide should not be affected to a clinically meaningful degree by interaction of concurrent medications with specific isozymes of CYP450 nor would administration of temozolomide alter by competitive inhibition the metabolism of other drugs.

Drug	Recommendation	Comments
Tepotinib (Tepmetko)	Contraindicated.<br>Use alternative COVID agent.<br>Do not use nirmatrelvir/ritonavir.	The mechanism for the predicted interaction is inhibition of the CYP3A4-mediated metabolism of tepotinib and inhibition of P-gp mediated efflux of tepotinib. Coadministration had no clinically significant effect on the pharmacokinetics of midazolam (sensitive CYP3A substrate).
Thalidomide	No interaction expected.	Coadministration has not been studied. A clinically significant pharmacokinetic interaction is unlikely as thalidomide is metabolized via non-enzymatic hydrolysis.
Trametinib	No interaction expected.	Coadministration has not been studied. A clinically significant pharmacokinetic interaction is unlikely as trametinib is metabolized via deacetylation (alone or with mono-oxygenation) or in combination with glucuronidation biotransformation pathways; ≥75% unchanged drug in plasma.
Trifluridine and Tipiraci	No interaction expected.	Coadministration has not been studied. A clinically significant pharmacokinetic interaction is unlikely as thalidomide is metabolized via non-CYP P450 pathways. Trifluridine, tipiracil and its metabolite FTY did not inhibit or induce CYP3A4, 1A2 or 2B6 in vitro studies.
Triptorelin	No interaction expected.	Coadministration has not been studied. A clinically significant pharmacokinetic interaction is unlikely as thalidomide is metabolized possibly via degradation by peptidases.
Tucatinib	Drug interaction not likely to be clinically relevant. Continue with standard dosing.	Tucatinib AUC was minimally changed when coadministered with itraconazole.
Umbralisib	Contraindicated (use within past 14 days).<br>Use alternative COVID agent.<br>Do not use nirmatrelvir/ritonavir.	Coadministration has not been studied. Umbralisib is a CYP3A4 inducer and may reduce concentrations of nirmatrelvir/ritonavir. Umbralisib inhibits CYP2C8, CYP2C9, CYP2C19, and CYP3A4, but does not inhibit CYP1A2, CYP2B6, and CYP2D6. Given long terminal half-life of umbralisib (~91 hrs), enzyme induction is expected to persist.
Vandetanib	Hold and restart 2 days after completing nirmatrelvir/ritonavir. Alternatively, monitor for vandetanib related toxicity if used concomitantly and interrupt therapy or consider dose reduction if necessary. Restart 2 days after completing nirmatrelvir/ritonavir.	Vandetanib AUC increased 1.1-fold when coadministered with itraconazole.

Drug	Recommendation	Comments
Vemurafenib	Hold and start nirmatrelvir/ritonavir 24 hours after the last vemurafenib dose. Alternatively, monitor for vemurafenib related toxicity if used concomitantly and interrupt therapy or consider dose reduction if necessary. Restart 2 days after completing nirmatrelvir/ritonavir.	Vemurafenib AUC increased 1.4-fold when coadministered with itraconazole. Terminal half-life ~57 hours.
Venetoclax (Venclexta)	Contraindicated.<br>Use alternative COVID agent.<br>Do not use nirmatrelvir/ritonavir.	Venetoclax AUC increased almost 8-fold when coadministered with ritonavir 50mg once daily. This effect is expected to be more pronounced with ritonavir 100mg twice daily. Concomitant use of strong CYP3A inhibitors, such as ritonavir, with venetoclax may increase the risk of tumor lysis syndrome.
Vinblastine	Vinblastine may be held in the context of acute infection. Restart vinblastine at least 2 days after completing nirmatrelvir/ritonavir.<br>Alternatively, vinblastine may be coadministered with close monitoring for hematologic and neurotoxicity. Some providers may wish to empirically reduce vinblastine dose, especially in patients who have previously experienced or are at high risk for toxicity.	Vinblastine AUC increased up to 1.6-fold when administered with boosted protease inhibitors. CYP3A4 inhibitors, including ritonavir, may increase the risk for autonomic and peripheral neurotoxicity and neutropenia.
Vincristine	Vincristine may be held in the context of acute infection. Restart vincristine no sooner than 2 days after completing nirmatrelvir/ritonavir. Alternatively, vincristine may be coadministered with close monitoring for hematologic and neurotoxicity. Some providers may wish to empirically reduce vincristine dose, especially in patients who have previously experienced or are at high risk for toxicity.	Decisions to hold or dose-adjust should be made in conjunction with the patient's oncologist.<br>Increased rates of hematologic toxicity and neuropathy (including autonomic neuropathy) have been reported with concomitant use of boosted protease inhibitors and vincristine.
Vismodegib	Drug interaction not likely to be clinically relevant. Continue with standard dosing.	Vismodegib is not an inducer of CYP1A2, CYP2B6 or CYP3A. Vismodegib is a substrate of CYP2C9, CYP3A4 and P-gp in vitro. Vismodegib AUC was minimally changed when coadministered with itraconazole.
Vorinostat	No interaction expected.	Coadministration has not been studied. A clinically significant pharmacokinetic interaction is unlikely as vorinostat is metabolized via glucuronidation (UGT2B17) and hydrolysis followed by B oxidation (non CYP-mediated).

Drug	Recommendation	Comments
Zanubrutinib	Hold and restart 2 days after completing nirmatrelvir/ritonavir. Alternatively, consider reducing zanubrutinib dose to 80 mg daily. Monitor for zanubrutinib related toxicity if used concomitantly and interrupt therapy or consider dose reduction if necessary. If grade 3 toxicity to zanubrutinib occurs (e.g., febrile neutropenia, thrombocytopenia with bleeding, neutropenia for 10 days or longer, etc.), interruption of zanubrutinib therapy should occur. Resume previous dose 2 days after completing nirmatrelvir/ritonavir.	Zanubrutinib AUC increased 3.8-fold when coadministered with itraconazole. Terminal half-life 2-4 hours.

AUC = Area under the curve; CYP = Cytochrome P450

Prepared by: Bassem Hamandi, BScPhm, PhD; Pamela Ng, BScPhm; Tessa Senneker, PharmD, ACPR2; Haiyue Tian.

Date last revised: April 1, 2022.

This document is intended for use by experienced clinicians, including prescribers and pharmacists. The information is not intended to replace sound professional judgment in individual situations, and should be used in conjunction with other reliable sources of information. Clinicians should always consider the risk/benefit profile for their individual patient, discuss these risks with the patient or caregiver before initiating therapy, and closely monitor for treatment benefit and adverse effects.

UNIVERSITY OF WATERLOO | SCHOOL OF PHARMACY

UNIVERSITY OF TORONTO
LESLIE DAN FACULTY OF PHARMACY

Updated: December 21, 2023

Nirmatrelvir/ Ritonavir (*Paxlovid™*)

What Prescribers and Pharmacists Need to Know

Why is nirmatrelvir/ritonavir used to treat COVID-19?

COVID-19 has an initial phase of viral replication and a significant inflammatory response in moderate illness. This inflammation can lead to poor outcomes, including hospitalization, invasive ventilation, and death. However, treatments that target SARS-CoV-2 replication, if administered before the inflammatory phase of COVID-19, can improve outcomes.

Nirmatrelvir works by binding to the SARS-CoV-2 3CL protease, which ultimately causes viral replication to stop. Ritonavir is a potent CYP3A4 inhibitor. It is not active against SARS-CoV-2 but is administered as a “boosting agent” to slow the metabolism of nirmatrelvir, thus increasing concentrations of nirmatrelvir.

Nirmatrelvir/ritonavir is a highly effective outpatient therapy based on available data, but **drug interactions must be managed (see Appendix)**. In a 2022 study, over 70% of 637 outpatients had at least 1 clinically significant drug-to-drug interaction (DDI) requiring mitigation.[1]

What is the benefit of nirmatrelvir/ritonavir for COVID-19?

The EPIC-HR study[2] has shown a benefit from treatment of adult outpatients with laboratory-proven SARS-CoV-2 infection who were not on supplemental oxygen and were within 5 days of symptom onset. The study suggests that nirmatrelvir/ritonavir may reduce the risk of hospitalization in these patients by 88%.

The initial research on nirmatrelvir/ritonavir was done in unvaccinated patients and prior to circulation of the Omicron variant. In recent real-world studies, timely use of Paxlovid™ reduced hospitalization, all-cause mortality, and visits to the emergency department in people who have moderate to severe weakened immune systems or who are older.[3,4]

Who should be considered for nirmatrelvir/ritonavir?

Nirmatrelvir/ritonavir should be considered for patients at higher risk of severe COVID-19 *(confirmed positive by PCR or rapid test),* and who are within 5 days of symptom onset.

PCR = polymerase chain reaction

Refer to your jurisdiction for eligibility criteria. Generally, patients who are good candidates are those who:

- Are older
- Have a weakened immune system
- Have serious health conditions
- Live in long-term care
- Are unvaccinated or under-vaccinated

Programs may also prioritize equity-deserving populations (e.g., racialized, living with a disability) and those living in rural communities.

Nirmatrelvir/ritonavir is recommended for people who are pregnant (if indicated) and can be offered to patients who are breastfeeding.[5]

Updated by: Alice Tseng, PharmD, FCSHP, AAHIVP; Claudia Bucci, BScPhm, PharmD, ACPR; Pierre Giguère, BPharm, MSc AAHIVP; Kelly Grindrod, PharmD; Heather Kertland, PharmD, FCSHP; Rosemary Killeen, BScPhm, PGCert, RPh; Todd Koch, BScPhm; Sharan Lail, BScPhm, PharmD, ACPR; Menaka Pai, MSc, MD, FRCPC; Tessa Senneker, PharmD, ACPR; Deborah Yoong, BScPhm, ACPR, PharmD.
Based on original document by: Alice Tseng, PharmD, FCSHP, AAHIVP; Claudia Bucci, BScPhm, PharmD, ACPR; Matthew Chow, BScPhm, PharmD, ACPR; Pierre Giguère, BPharm, MSc AAHIVP; Kelly Grindrod, PharmD; Bassem Hamandi, BScPhm, PhD; Heather Kertland, PharmD, FCSHP; Rosemary Killeen, BScPhm, PGCert, RPh; Todd Koch, BScPhm; Adam S. Komorowski, MD; Sharan Lail, BScPhm, PharmD, ACPR; Kori Leblanc, BScPhm, PharmD, ACPR; Andrew Morris, MD, FRCPC; Christine Papoushek, PharmD; Linda Robinson, BSc.Phm, AAHIVP; Tessa Senneker, PharmD, ACPR; Deborah Yoong, BScPhm, ACPR, PharmD; Beth Leung, PharmD, MSCI, BCPS AQID.
Design by: Adrian Poon, BA.

How do I dose nirmatrelvir/ritonavir for treatment of COVID-19?

1. Paxlovid™ consists of 2 drugs packaged together:
   - Nirmatrelvir (pink) 150 mg tablet
   - Ritonavir (white) 100 mg tablet
2. Each carton contains 5 blister cards. One blister card is used each day. The full course of treatment is 5 days.
3. Take 2 pink tablets of nirmatrelvir and 1 white tablet of ritonavir (3 tablets total) together at the same time, once in the morning and once in the evening for 5 days (i.e., 6 tablets per day).
   - May be taken with or without food.
   - May be split or crushed and mixed with common food:
     ↳ For guidance, see "Crushing Nirmatrelvir/ritonavir".

Special Dosing Considerations:

eGFR† 30 to 59 mL/min:
Nirmatrelvir 150 mg and ritonavir 100 mg taken together orally BID x 5 days.

eGFR† <30 mL/min:[6]
Day 1: Nirmatrelvir 300 mg and ritonavir 100 mg
Day 2 to 5: Nirmatrelvir 150 mg and ritonavir 100 mg once daily.

Dialysis: Dose for eGFR <30 mL/min; give after dialysis.

If dialysis and weight <40 kg:
Nirmatrelvir 150 mg and ritonavir 100 mg q48h x 3 doses; give after dialysis.

Severe hepatic impairment (Child-Pugh Class C):
Nirmatrelvir/ritonavir is not recommended.

†eGFR = estimated glomerular filtration rate

What side effects should I be aware of?

Common side effects of nirmatrelvir/ritonavir are generally mild and can include dysgeusia (taste disturbance), diarrhea, hypertension, myalgia, vomiting and headache.

Paxlovid™ product monograph

https://covid-vaccine.canada.ca/info/pdf/paxlovid-pm-en.pdf

What drug interactions should I consider before prescribing nirmatrelvir/ritonavir?

- **Ritonavir is a potent inhibitor of CYP3A4 isoenzyme and various drug transporters (e.g., P-glycoprotein).**
  - Onset of ritonavir inhibition is rapid and takes a few days to dissipate after completion of therapy.
- **Ritonavir and nirmatrelvir are both CYP3A4 substrates.**
- **Nirmatrelvir/ritonavir is contraindicated in patients taking drugs that are:**
  - Highly metabolized by CYP3A4 where elevated concentrations can be life-threatening.
  - Potent CYP3A4 inducers which may reduce the effectiveness of nirmatrelvir/ritonavir and contribute to the development of drug resistance.

What if my patient is taking therapy for human immunodeficiency virus (HIV)?

Patients taking ritonavir or cobicistat for HIV therapy should continue their complete antiretroviral regimen at usual dosing while taking nirmatrelvir/ritonavir.

Nirmatrelvir/ritonavir has many drug interactions. See page 3 →

What if my patient is taking a drug that interacts with nirmatrelvir/ritonavir?

- If the patient is taking or has taken a **CYP3A4 enzyme inducer** in the last 14 days (e.g., certain anticonvulsants, antineoplastics, a rifamycin, St. John's wort): Do NOT prescribe nirmatrelvir/ritonavir.
- ▲ If the patient takes an interacting drug with a **long plasma half-life and narrow therapeutic window** (e.g., certain antiarrhythmics, antipsychotics, antineoplastics), the interacting drug will persist in the body after the last dose and may still interact with nirmatrelvir/ritonavir: Do NOT prescribe nirmatrelvir/ritonavir even if the interacting drug can be held.
- ● If the patient takes an interacting drug that can be held, hold the drug starting the first day of nirmatrelvir/ritonavir therapy, and resume 2 days after the last dose of nirmatrelvir/ritonavir treatment.
- ◆ A specialist prescriber or pharmacist may be able to help adjust the dose or dosing interval, replace the drug with an alternative agent, manage side effects, and guide therapeutic drug monitoring.

1 Leung V, et al. (2023) A retrospective cohort study of prescribing outcomes in outpatients treated with nirmatrelvir–Ritonavir for COVID-19 in an interdisciplinary community clinic. *PLoS ONE*. 18(10): e0293302. https://doi.org/10.1371/journal.pone.0293302
2 Hammond J, Leister-Tebbe H, Gardner A, Abreu P et al. Oral Nirmatrelvir for High-Risk, Nonhospitalized Adults with Covid-19. *NEJM*. doi: 10.1056/NEJMoa2118542
3 Najjar-Debbiny R, et al. Effectiveness of Paxlovid in Reducing Severe Coronavirus Disease 2019 and Mortality in High-Risk Patients. *Clin Infect Dis*. 2023 Feb 8;76(3):e342-e349. doi: 10.1093/cid/ciac443
4 Dormuth CR, Kim JD, Fisher A, Piszczek J, Kuo IF. Nirmatrelvir-Ritonavir and COVID-19 Mortality and Hospitalization Among Patients With Vulnerability to COVID-19 Complications. *JAMA Netw Open*. 2023;6(10):e2336678. doi:10.1001/jamanetworkopen.2023.36678
5 COVID-19 Treatment Guidelines Panel. Coronavirus Disease 2019 (COVID-19) Treatment Guidelines. National Institutes of Health. Available at https://www.covid19treatmentguidelines.nih.gov/. Accessed December 19, 2023.
6 Hiremath S, McGuinty M, Argyropoulos C et al. Prescribing nirmatrelvir/ritonavir for COVID-19 in advanced CKD. *Clin J Am Soc Nephrol.* 2022;17(8):1247-50. https://pubmed.ncbi.nlm.nih.gov/35680135/

Nirmatrelvir/Ritonavir (*Paxlovid*™) Drug Interactions:

This is not an exhaustive list. Consultation with a pharmacist who can obtain a complete medication, recreational, and natural health product history from the patient is recommended prior to prescribing nirmatrelvir/ritonavir.

In certain instances, extended duration therapy (≥10 days) of Paxlovid™ may be considered. With more prolonged treatment, drug interaction severity and/or management strategies may be different. Prescribers who intend to use Paxlovid™ beyond the standard 5-day duration are encouraged to refer to the Liverpool COVID-19 Interaction Checker (see green button below) and select the "Nirmatrelvir/ritonavir (extended administration; 10 days or longer)" option for specific drug interaction guidance.

Symbol	Severity	Recommendation	Rationale
▲<br>△	Contraindicated<br>Contraindicated (use within past 14 days)	Use alternative COVID agent. **Do not use nirmatrelvir/ritonavir.**	Stopping the drug will not mitigate the interaction (e.g., prolonged half-life, narrow therapeutic index, prolonged enzyme-inducing effects which may decrease effectiveness of nirmatrelvir/ritonavir). Do not coadminister due to risk of serious toxicity.
●	Do not coadminister	Hold and restart 2 days after completing nirmatrelvir/ritonavir.	Significant ↑ in drug concentrations expected. Do not coadminister due to risk of serious toxicity.
◆	Caution	Therapy modification required (see Appendix).	Significant ↑/↓ in drug concentrations expected, which may lead to serious toxicity or impaired efficacy. Only coadminister if the interacting drug can be safely held or dose-adjusted and closely monitored (see Appendix). Expert consultation may be useful.
✔	Drug interaction not likely to be clinically relevant	Continue with standard dosing.	Although mentioned in the monograph, clinically relevant interaction is not anticipated (e.g., minimal impact on certain metabolic pathways, wide therapeutic index, and short course of nirmatrelvir/ritonavir).

- ◆ Abemaciclib (*Verzenio*)
- ● Alfuzosin (*Xatral*)
- ● Aliskiren (*Rasilez*)
- ◆ Alprazolam (*Xanax*)
- ▲ Amiodarone
- ✔ Amitriptyline
- ◆ Amlodipine (*Norvasc*)
- △ Apalutamide (*Erleada*)
- ◆ Apixaban (*Eliquis*)
- ◆ Aripiprazole (*Abilify*), oral
- ◆ Atorvastatin (*Lipitor*)
- ✔ Atovaquone
- ▲ Bosentan (*Tracleer*)
- ● Bosutinib (*Bosulif*)
- ◆ Brexpiprazole (*Rexulti*)
- ✔ Budesonide
- ✔ Bupropion
- ◆ Buspirone (*Buspar*)
- △ Carbamazepine (*Tegretol*)
- △ Cariprazine (*Vraylar*)
- ◆ Ceritinib (*Zykadia*)
- ● Cisapride
- ✔ Citalopram
- ✔ Clarithromycin
- ✔ Clomipramine
- ● Clonazepam
- ◆ Clopidogrel (*Plavix*)
- ● Clorazepate
- ▲ Clozapine (*Clozaril*)
- ● Cobimetinib (*Cotellic*)
- ● Colchicine in renal/hepatic impairment
- ◆ Cyclosporine (*Neoral*)
- ◆ Dabigatran
- △ Dabrafenib (*Tafinlar*)
- ◆ Darifenacin (*Enablex*)
- ◆ Dasatinib (*Sprycel*)
- ✔ Desipramine
- ◆ Dexamethasone, high dose
- ● Diazepam (*Valium*)
- ◆ Digoxin
- ◆ Diltiazem (*Tiazac, Cardizem*)
- ● Disopyramide (*Rythmodan*)
- ✔ Divalproex
- ● Dofetilide
- ✔ Dronabinol
- ▲ Dronedarone (*Multaq*)
- ◆ Edoxaban (*Lixiana*)
- ◆ Elagolix (*Orilissa*)
- ▲ Eletriptan (*Relpax*)
- ◆ Elexacaftor/tezacaftor/ ivacaftor (*Trikafta*)
- ◆ Encorafenib (*Braftovi*)
- △ Enzalutamide
- ● Eplerenone (*Inspra*)
- ● Ergot alkaloids (e.g., dihydroergotamine, ergonovine)
- ◆ Erythromycin
- △ Eslicarbazepine
- ✔ Ethinyl estradiol
- ● Everolimus (*Certican*)
- ◆ Felodipine
- ▲ Fentanyl (*Duragesic*)
- ● Finerenone (*Kerendia*)
- ▲ Flecainide
- ● Flibanserin (*Addyi*)
- ✔ Fluoxetine
- ● Flurazepam
- ✔ Fluticasone (*Flovent, Flonase*)
- ✔ Fluvoxamine
- ◆ Fostamatinib (*Tavalisse*)
- ✔ Fusidic acid, topical
- ● Glecaprevir/Pibrentasvir (*Maviret*)
- ◆ Hydrocodone
- ● Ibrutinib (*Imbruvica*)
- ✔ Imipramine
- ◆ Isavuconazole (*Cresemba*)
- ✔ Itraconazole
- △ Ivabradine (*Lancora*)
- ◆ Ivacaftor (*Kalydeco*)
- △ Ivosidenib (*Tibsovo*)
- ✔ Ketoconazole
- ✔ Lamotrigine
- ● Lomitapide (*Juxtapid*)
- △ Lorlatinib (*Lorbrena*)
- ● Lovastatin
- △ Lumacaftor/ivacaftor (*Orkambi*)
- ▲ Lurasidone (*Latuda*)
- ✔ Maprotiline
- ✔ Maraviroc
- ● Meperidine (*Demerol*)
- ✔ Methamphetamine
- ✔ Metoprolol
- ● Midazolam, oral
- △ Mitotane (*Lysodren*)
- ◆ Modafinil
- ● Naloxegol (*Movantik*)
- ● Neratinib (*Nerlynx*)
- ◆ Nifedipine
- ◆ Nilotinib (*Tasigna*)
- ● Nitrazepam (*Mogadon*)
- ✔ Nortriptyline
- △ Oxcarbazepine
- ◆ Oxycodone (*Percocet, OxyNEO*)
- ✔ Paroxetine
- △ Phenobarbital
- △ Phenytoin (*Dilantin*)
- ▲ Pimozide
- ✔ Prednisone
- △ Primidone
- ▲ Propafenone
- ◆ Quetiapine (*Seroquel*)
- ▲ Quinidine
- ● Quinine
- ✔ Raltegravir
- ▲ Ranolazine (*Corzyna*)
- ◆ Rifabutin
- △ Rifampin
- △ Rifapentine
- ▲ Riociguat (*Adempas*)
- ◆ Risperidone (*Risperdal*), oral
- ▲ Risperidone, long-acting injection (*Risperdal Consta*)
- ● Rivaroxaban (*Xarelto*)
- ◆ Rosuvastatin (*Crestor*)
- ● Salmeterol (*Serevent, Advair*)
- ◆ Saxagliptin (*Onglyza*)
- ✔ Sertraline
- ◆ Sildenafil for ED† (*Viagra*)
- ▲ Sildenafil for PAH‡ (*Revatio*)
- ● Silodosin (*Rapaflo*)
- ● Simvastatin
- ● Sirolimus (*Rapamune*)
- ● Sonidegib (*Odomzo*)
- △ St. John's wort (*Hypericum perforatum*)
- ● Tacrolimus (*Prograf, Advagraf, Envarsus*)
- ◆ Tadalafil for ED† (*Cialis*)
- ▲ Tadalafil for PAH‡ (*Adcirca*)
- ◆ Tamsulosin (*Flomax*)
- ▲ Tepotinib (*Tepmetko*)
- ◆ Tezadaftor/ivacaftor (*Symdeko*)
- ✔ Theophylline
- ● Ticagrelor (*Brilinta*)
- ✔ Timolol
- ◆ Tofacitinib (*Xeljanz*)
- ▲ Tolvaptan (*Jinarc, Samsca*)
- ◆ Tramadol
- ◆ Trazodone (*Desyrel*)
- ✔ Triamcinolone
- ● Triazolam (*Halcion*)
- ✔ Trimipramine
- ● Ubrogepant (*Ubrelvy*)
- ◆ Upadacitinib (*Rinvoq*)
- ● Vardenafil (*Levitra*) for ED†
- ▲ Vardenafil (*Levitra*) for PAH‡
- ▲ Venetoclax (*Venclexta*)
- ✔ Venlafaxine
- ◆ Verapamil
- ◆ Vinblastine
- ◆ Vincristine
- △ Vorapaxar (*Zontivity*)
- ◆ Voriconazole (*Vfend*)
- ◆ Warfarin
- ◆ Ziprasidone (*Zeldox*)
- ◆ Zolpidem (*Sublinox, Ambien*)
- ◆ Zopiclone (*Imovane*)

Liverpool's COVID-19 Interaction Checker
https://www.covid19-druginteractions.org/

University Health Network & Kingston Health Sciences Centre Paxlovid-Oncology DDI
https://hivclinic.ca/wp-content/uploads/2023/12/Oncology-Related-Paxlovid-Drug-Interactions.pdf

†ED = erectile dysfunction ‡PAH = pulmonary arterial hypertension

This document is intended for use by experienced clinicians, including prescribers and pharmacists. The information is not intended to replace sound professional judgment in individual situations, and should be used in conjunction with other reliable sources of information. Clinicians should always consider the risk/benefit profile for their individual patient, discuss these risks with the patient or caregiver before initiating therapy, and closely monitor for treatment benefit and adverse effects.

Appendix: Nirmatrelvir/ritonavir (*Paxlovid*™) Drug Interactions

Guiding principles for managing drug interactions categorized as 🔴 and 🔶.

There is limited drug interaction data for nirmatrelvir/ritonavir (which is a potent CYP3A4/P-glycoprotein inhibitor). Most potential interactions listed below are based on known/anticipated effects with ritonavir alone or with other protease inhibitors. In some instances, pharmacokinetic interaction data for other potent CYP3A4 inhibitors (e.g., clarithromycin, ketoconazole) are included in this table to help predict the potential extent of an interaction effect with nirmatrelvir/ritonavir.

This document should only be used in regards to a 5-day therapy for Paxlovid™ as drug interaction severity and/or management strategies may be different. Prescribers who intend to use Paxlovid™ beyond the standard 5-day duration are encouraged to refer to the Liverpool COVID-19 Interaction Checker (see green button on page 3) and select the "Nirmatrelvir/ritonavir (extended administration; 10 days or longer)" option for specific drug interaction guidance.

General recommendation for 🔴 🔶 drugs:
Hold the interacting drug for one week (i.e., beginning on the first day of nirmatrelvir/ritonavir and resuming two days after completing nirmatrelvir/ritonavir).

› *Ritonavir inhibition is not immediately reversible.*

If holding a drug for one week is not a safe option:

- Use an alternative COVID-19 agent for 🔴 drugs, or;
- Consider therapy modification for 🔶 drugs.

Caution: ⚠

Some drugs may need to be held longer due to a greater sensitivity to ritonavir inhibition (e.g., calcineurin inhibitors).

In many instances, replacing a drug is not feasible, and may introduce more risk of harm or error (e.g., patient takes both the held and new drug, forgets to restart original drug, etc).

› Recommendations in this appendix are based on Canadian product monographs, the Liverpool COVID-19 Drug Interactions Database (University of Liverpool, 2023), Lexi-Interact Online Database (Hudson OH, Wolters Kluwer, 2023), and additional references as noted.

Disclaimer

This document is intended for use by experienced clinicians, including prescribers and pharmacists. The information is not intended to replace sound professional judgment in individual situations, and should be used in conjunction with other reliable sources of information. Clinicians should always consider the risk/benefit profile for their individual patient, discuss these risks with the patient or caregiver before initiating therapy, and closely monitor for treatment benefit and adverse effects.

Neither the University of Waterloo, the University of Toronto, nor the authors and their respective institutions are responsible for deletions or inaccuracies in information or for claims of injury resulting from any such deletions or inaccuracies. Mention of specific drugs, drug doses, or drug combinations within this document does not constitute endorsement by the University of Waterloo, the University of Toronto, or the authors and their respective institutions.

We acknowledge the Ontario COVID-19 Science Advisory Table for their collaboration on the original document.

	Drug	Recommendation	Comments
🔶	Abemaciclib (*Verzenio*)	Hold and restart 2 days after completing nirmatrelvir/ritonavir.<br>Alternatively, for patients who have not previously had dose reduction for toxicity, consider a dose reduction to 50 mg once daily with close monitoring for toxicity.	Decisions to hold or dose-adjust should be made in conjunction with the patient's oncologist.<br>Cyclin-dependent kinase inhibitors are generally held for acute infection. Abemaciclib AUC increased over 3-fold when coadministered with clarithromycin.
🔴	Alfuzosin (*Xatral*)	Hold and restart 2 days after completing nirmatrelvir/ritonavir.<br>Alternatively, may consider giving every other day in patients with heightened risk of urinary retention. Monitor for hypotension.	Alfuzosin AUC increased 3-fold when coadministered with ketoconazole 400 mg.
🔴	Aliskiren (*Rasilez*)	Hold and restart 2 days after completing nirmatrelvir/ritonavir.	Aliskiren AUC increased 5 to 6-fold when coadministered with itraconazole.
🔶	Alprazolam (*Xanax*)	Hold and restart 2 days after completing nirmatrelvir/ritonavir.<br>Alternatively, reduce alprazolam dose by at least 50% and monitor for increased effects.	Alprazolam AUC increased 148% and half-life increased from 13 to 30 hours when coadministered with ritonavir 200 mg x 4 doses.

AUC = Area under the curve

Appendix (Page 2)

Drug	Recommendation	Comments
◆ Amlodipine (*Norvasc*)	Reduce amlodipine dose by 50% or take dose every other day. Restart usual dose 2 days after completing nirmatrelvir/ritonavir.<br><br>Monitor blood pressure. May consider continuing with usual dosing in patients at low risk of bradycardia or hypotension.	Amlodipine AUC increased 2-fold when coadministered with indinavir/ritonavir or paritaprevir/ritonavir.
◆ Apixaban (*Eliquis*)	**If possible, use alternative COVID-19 agent. If not possible, ensure stable renal function, then:**<br><br>A) If already on low dose (2.5 mg BID) apixaban, continue.<br><br>B) If acute venous thromboembolism (VTE):<br>⚠ Low risk of clot:<br>Hold apixaban. 12 hours after the last dose of apixaban, start nirmatrelvir/ritonavir AND aspirin 81 mg daily. Finish aspirin 1 day after completing nirmaltrevir/ritonavir. Restart apixaban 2 days after completing nirmatrelvir/ritonavir.<br><br>⚠ High risk of clot:<br>Hold apixaban. 12 hours after the last dose of apixaban, start nirmatrelvir/ritonavir AND therapeutic dosing of a subcutaneous low molecular weight heparin (LMWH) such as:<br>◦ Dalteparin: 200 units/kg daily OR 100 units/kg every 12 hours if >90 kg;<br>◦ Enoxaparin: 1 mg/kg every 12 hours (preferred) OR 1.5 mg/kg once every 24 hours;<br>◦ Tinzaparin: 175 anti-Xa units/kg once daily.<br><br>Finish LMWH 1 day after completing nirmatrelvir/ritonavir. Restart apixaban 2 days after completing nirmatrelvir/ritonavir.<br><br>C) If atrial fibrillation:<br>Decrease apixaban to 2.5 mg BID. Resume usual dose 2 days after completing nirmatrelvir/ritonavir.<br><br>See *Paxlovid for a Patient on a DOAC* for more details. https://uwaterloo.ca/pharmacy/sites/default/files/uploads/documents/paxlovid_for_a_patient_on_a_doac_0.pdf	Canadian product monograph states that coadministration with ritonavir is contraindicated. However, US product monograph suggests to decrease 5 mg twice daily dose to 2.5 mg twice daily when combined with strong inhibitors of CYP3A4 and P-glycoprotein.<br>Eliquis (U.S.) Prescribing Information. Accessed February 8, 2022. https://www.accessdata.fda.gov/drugsatfda_docs/label/2012/202155s000lbl.pdf<br><br>Observational data from the US found no increased risk of bleeding in those prescribed apixaban, rivaroxaban, and dabigatran with nirmatrelvir/ritonavir, even when recommended dosing adjustments were not followed.<br>Vazquez S, Wilson A, Witt D. Management of potential drug-drug interactions with nirmatrelvir-ritonavir and oral anticoagulants: a case series. *J Thromb Thrombolysis*. 2022;54:583–86. https://www.ncbi.nlm.nih.gov/pmc/articles/PMC9579540/<br><br>⚠ **High risk of clot includes:**<br>◦ Clot within past 6 months<br>◦ Clot at any time in past when anticoagulation interrupted<br>◦ Active cancer with clot at any point in cancer journey<br>◦ Diagnosis of antiphospholipid antibody syndrome
◆ Aripiprazole (*Abilify*), oral	Reduce aripiprazole oral dose by 50% and resume usual dose 2 days after completing nirmatrelvir/ritonavir.<br><br>Monitor for confusion, restlessness, and sedation.	Aripiprazole AUC increased almost 2-fold when coadministered with ketoconazole.<br><br>No clinically relevant interaction expected with long-acting injection.
◆ Atorvastatin	Hold and restart 2 days after completing nirmatrelvir/ritonavir.<br><br>Alternatively, reduce atorvastatin to the equivalent of 10 mg daily by alternative day dosing, cutting the tablet, or obtaining a new prescription. Resume usual dose 2 days after completing nirmatrelvir/ritonavir.	Atorvastatin AUC increased almost 6-fold when coadministered with lopinavir/ritonavir 400/100 mg twice daily.
● Bosutinib (*Bosulif*)	Hold bosutinib and start nirmatrelvir/ritonavir 24 hours after the last bosutinib dose. Restart bosutinib 2 days after completing nirmatrelvir/ritonavir.	Decisions to hold or dose-adjust should be made in conjunction with the patient's oncologist.<br><br>Bosutinib AUC increased almost 9-fold when coadministered with ketoconazole.

This document is intended for use by experienced clinicians, including prescribers and pharmacists. The information is not intended to replace sound professional judgment in individual situations, and should be used in conjunction with other reliable sources of information. Clinicians should always consider the risk/benefit profile for their individual patient, discuss these risks with the patient or caregiver before initiating therapy, and closely monitor for treatment benefit and adverse effects.

AUC = Area under the curve

Appendix (Page 3)

	Drug	Recommendation	Comments
◆	Brexpiprazole (*Rexulti*)	Reduce brexpiprazole dose by 50% and resume usual dose 2 days after completing nirmatrelvir/ritonavir.<br>Monitor for confusion, restlessness, sedation.	Brexpiprazole AUC increased 97% when coadministered with ketoconazole.
◆	Buspirone (*Buspar*)	Hold and restart 2 days after completing nirmatrelvir/ritonavir.<br>Alternatively, reduce buspirone dose to 2.5 mg daily if the usual dose is 20 to 30 mg/day.	Buspirone AUC increased 19-fold when coadministered with itraconazole 200 mg/day for 4 days.
◆	Ceritinib (*Zykadia*)	Hold and restart 2 days after completing nirmatrelvir/ritonavir.<br>Alternatively, consider reducing ceritinib dose by 33% and monitor for toxicity.	Canadian product monograph recommends to avoid concomitant use. However, US product monograph suggests reducing dose by 33%, rounded to nearest 150 mg dosage strength.<br>Zykadia (U.S.) Prescribing Information. Accessed February 8, 2022. https://www.accessdata.fda.gov/drugsatfda_docs/label/2019/205755s016lbl.pdf<br>Decision to hold or dose-adjust ceritinib should be made in conjunction with the patient's oncologist.<br>Ceritinib AUC increased 3-fold when single dose coadministered with ketoconazole.
●	Cisapride	Hold and restart 2 days after completing nirmatrelvir/ritonavir.	Potential for serious and/or life-threatening adverse effects, including cardiac arrhythmias.
●	Clonazepam	Hold and restart 2 days after completing nirmatrelvir/ritonavir.<br>If an anxiolytic is needed, use lorazepam, oxazepam, or temazepam at usual doses.	Due to prolonged benzodiazepine half-life, coadministration is not recommended.
◆	Clopidogrel (*Plavix*)	**Acute coronary syndrome (ACS)/percutaneous coronary intervention (PCI):**<br>• If <1 month since ACS: Use alternative COVID-19 agent.<br>• If <3 months since ACS or <1 month since PCI (no ACS): Consider switching clopidogrel to prasugrel (if age <75, weight >60 kg, and no history of stroke/TIA) and resume clopidogrel 2 days after completing nirmatrelvir/ritonavir;<br>• If >3 months since ACS or >1 month since PCI (no ACS): Continue clopidogrel with acetylsalicylic acid (ASA) during nirmatrelvir/ritonavir therapy. If not taking ASA, consider switching to prasugrel (if age <75, weight >60 kg, and no history of stroke/TIA) and resume clopidogrel 2 days after completing nirmatrelvir/ritonavir.	Coadministration will decrease the antiplatelet effect of clopidogrel.<br>Clopidogrel active metabolite AUC decreased by 51 to 69% when coadministered with ritonavir.
●	Clorazepate	Hold and restart 2 days after completing nirmatrelvir/ritonavir.<br>If an anxiolytic is needed, use lorazepam, oxazepam, or temazepam at usual doses.	Due to prolonged benzodiazepine half-life, coadministration is not recommended.
●	Cobimetinib (*Cotellic*)	Hold cobimetinib and start nirmatrelvir/ritonavir 24 hours after the last cobimetinib dose. Restart cobimetinib 2 days after completing nirmatrelvir/ritonavir.	Decisions to hold or dose-adjust should be made in conjunction with the patient's oncologist.<br>Cobimetinib AUC increased almost 7-fold when coadministered with ketoconazole.
●	Colchicine in renal/hepatic impairment	*Next page*	*Next page*

This document is intended for use by experienced clinicians, including prescribers and pharmacists. The information is not intended to replace sound professional judgment in individual situations, and should be used in conjunction with other reliable sources of information. Clinicians should always consider the risk/benefit profile for their individual patient, discuss these risks with the patient or caregiver before initiating therapy, and closely monitor for treatment benefit and adverse effects.

TIA = Transient ischemic attack
AUC = Area under the curve

Appendix (Page 4)

Drug	Recommendation	Comments
Colchicine in renal/ hepatic impairment	**Coadministration is contraindicated in patients with renal and/or hepatic impairment.**<br><br>In patients with <u>normal renal/hepatic function,</u> colchicine may be administered at a lowered dose if practical:<br>• Treatment of gout flares: 0.6 mg x 1 dose, then 0.3 mg (½ tablet) 1 hour later. Repeat dose no earlier than 3 days.<br>• Prevention of gout flares:<br>a) If on 0.6 mg twice daily: decrease to 0.3 mg once daily;<br>b) If on 0.3 mg twice daily: decrease to 0.3 mg once every 2 days.<br>• Treatment of Familial Mediterranean fever: Maximum 0.6 mg (or 0.3 mg twice daily).<br><br>In all cases, resume usual colchicine dose 2 days after completing nirmatrelvir/ritonavir.	Drug interaction could lead to potentially life-threatening/fatal adverse events.
Cyclosporine (*Neoral*)	Decision to start nirmatrelvir/ritonavir should be done in conjunction with the patient's transplant provider.<br><br>Reduce cyclosporine total daily dose by 80% and start nirmatrelvir/ritonavir 12 hours after the last cyclosporine dose. Continue at reduced dose throughout nirmatrelvir/ritonavir therapy.<br><br>Resuming transplant immunotherapy after the last dose of nirmatrelvir/ritonavir should be guided by therapeutic drug monitoring and in conjunction with the patient's transplant provider.	Check cyclosporine concentrations 2 days after the last dose of nirmatrelvir/ritonavir.<br>• If subtherapeutic/therapeutic: Resume at 100% of baseline dose.<br>• If supratherapeutic: Continue at 20% of baseline dose for 2 days, then 100% of baseline dose thereafter.<br><br>Repeat cyclosporine at weekly intervals as needed.<br><br>Giguère P, Deschenes MJ, Van Loon M et al. Management and outcome of COVID-19 infection using nirmaltrevir/ritonavir in kidney transplant patients. *Clin J Am Soc Nephron*. 2023;18:913-919. https://doi.org/10.2215/cjn.0000000000000186
Dabigatran	**If possible, use alternative COVID-19 agent. If not possible, then:**<br><br>A) If already on low dose (110 mg BID) dabigatran, continue.<br><br>B) If acute venous thromboembolism (VTE):<br>⚠ <u>Low risk of clot:</u><br>Hold dabigatran. 12 hours after the last dose of dabigatran, start nirmatrelvir/ritonavir <u>AND</u> aspirin 81 mg daily. Finish aspirin 1 day after completing nirmatrelvir/ritonavir. Restart dabigatran 2 days after completing nirmaltrevir/ritonavir.<br><br><u>High risk of clot:</u><br>⚠ Hold dabigatran. 12 hours after the last dose of dabigatran, start nirmatrelvir/ritonavir <u>AND</u> therapeutic dosing of a subcutaneous low molecular weight heparin (LMWH) such as:<br>◦ Dalteparin: 200 units/kg daily <u>OR</u> 100 units/kg every 12 hours if >90 kg;<br>◦ Enoxaparin: 1 mg/kg every 12 hours (preferred) <u>OR</u> 1.5 mg/kg once every 24 hours;<br>◦ Tinzaparin: 175 anti-Xa units/kg once daily.<br><br>Finish LMWH 1 day after completing nirmatrelvir/ritonavir. Restart dabigatran 2 days after completing nirmatrelvir/ritonavir.<br><br>C) If atrial fibrillation:<br>Decrease dabigatran to 110 mg BID (if eGFR >50 mL/minute) <u>OR</u> decrease to 75 mg BID (if eGFR 30 to 50 mL/minute). Resume usual dose 2 days after completing nirmatrelvir/ritonavir.	Dabigatran AUC increased almost 2-fold when coadministered with nirmatrelvir/ritonavir.<br><br>Observational data from the US found no increased risk of bleeding in those prescribed apixaban, rivaroxaban, and dabigatran with nirmatrelvir/ritonavir, even when recommended dosing adjustments were not followed.<br><br>Vazquez S, Wilson A, Witt D. Management of potential drug-drug interactions with nirmatrelvir-ritonavir and oral anticoagulants: a case series. *J Thromb Thrombolysis*. 2022;54:583–86. https://www.ncbi.nlm.nih.gov/pmc/articles/PMC9579540/<br><br>⚠ **High risk of clot includes:**<br>◦ Clot within past 6 months<br>◦ Clot at any time in past when anticoagulation interrupted<br>◦ Active cancer with clot at any point in cancer journey<br>◦ Diagnosis of antiphospholipid antibody syndrome<br><br>See *Paxlovid for a Patient on a DOAC* for more details. https://uwaterloo.ca/pharmacy/sites/default/files/uploads/documents/paxlovid_for_a_patient_on_a_doac_0.pdf

This document is intended for use by experienced clinicians, including prescribers and pharmacists. The information is not intended to replace sound professional judgment in individual situations, and should be used in conjunction with other reliable sources of information. Clinicians should always consider the risk/benefit profile for their individual patient, discuss these risks with the patient or caregiver before initiating therapy, and closely monitor for treatment benefit and adverse effects.

AUC = Area under the curve

Appendix (Page 5)

Drug	Recommendation	Comments
◆ Darifenacin (*Enablex*)	Reduce dose to 7.5 mg darifenacin and resume usual dose 2 days after completing nirmatrelvir/ritonavir.	Darifenacin AUC increased 5-fold when coadministered with ketoconazole.
◆ Dasatinib (*Sprycel*)	**Chronic phase chronic myelogenous leukemia (CML):** Hold and restart 2 days after completing nirmatrelvir/ritonavir. Alternatively, consider reducing dasatinib dose to 20 to 40 mg and monitor for toxicity.<br><br>**Accelerated or blast phase CML:** Do not coadminister; use alternate COVID-19 therapy.	Decisions to hold or dose-adjust dasatinib should be made in conjunction with the patient's oncologist.<br><br>Dasatinib AUC increased 5-fold when coadministered with ketoconazole.
◆ Dexamethasone, high dose	**High dose (≥20 mg daily):** Reduce dexamethasone dose by 50% and resume usual dose 2 days after completing nirmatrelvir/ritonavir.<br><br>**Low dose (<20 mg daily):** Continue with usual dose during nirmatrelvir/ritonavir.	Dexamethasone AUC increased almost 3-fold when coadministered with voriconazole. Li M, Zhu L, Chen L et al. Assessment of drug-drug interactions between voriconazole and glucocorticoids. *J Chemother*. 2018;30(5):296-303. https://doi.org/10.1080/1120009x.2018.1506693<br><br>Potential for risk of dexamethasone toxicity with high doses (≥20 mg daily).<br><br>Clinically significant interaction is not expected with dexamethasone at low doses, including when used for COVID-19 treatment.
● Diazepam (*Valium*)	Hold and restart 2 days after completing nirmatrelvir/ritonavir.<br><br>If an anxiolytic is needed, use lorazepam, oxazepam, or temazepam at usual doses.	Due to prolonged benzodiazepine half-life, coadministration is not recommended.
◆ Digoxin	Reduce digoxin dose by 50% **OR** hold and restart 2 days after completing nirmatrelvir/ritonavir.	
◆ Diltiazem (*Tiazac, Cardizem*)	Reduce diltiazem dose by 50% or take dose every other day. Restart usual dose 2 days after completing nirmatrelvir/ritonavir.<br><br>Monitor heart rate and blood pressure. May consider continuing with usual dosing in patients at low risk of bradycardia or hypotension.	Concentrations of calcium channel blockers are expected to increase when coadministered with nirmatrelvir/ritonavir.
● Disopyramide (*Rythmodan*)	Hold disopyramide and restart 2 days after completing nirmatrelvir/ritonavir.	Disopyramide is partially (25%) metabolized by CYP3A4, with 50% eliminated unchanged in the urine. Coadministration may lead to increased disopyramide levels.
● Dofetilide	If possible, use alternative COVID-19 agent.<br><br>Alternatively, hold dofetilide and restart 2 days after completing nirmatrelvir/ritonavir.	Dofetilide is metabolized to a small extent through CYP3A4.
◆ Edoxaban (*Lixiana*)	*Next page*	*Next page*

This document is intended for use by experienced clinicians, including prescribers and pharmacists. The information is not intended to replace sound professional judgment in individual situations, and should be used in conjunction with other reliable sources of information. Clinicians should always consider the risk/benefit profile for their individual patient, discuss these risks with the patient or caregiver before initiating therapy, and closely monitor for treatment benefit and adverse effects.

AUC = Area under the curve

Appendix (Page 6)

Drug	Recommendation	Comments
Edoxaban (*Lixiana*)	**If possible, use alternative COVID-19 agent. If not possible, ensure stable renal function, then:**<br>A) If already on low dose (30 mg once daily) edoxaban, continue.<br>B) If acute venous thromboembolism (VTE):<br>⚠ Low risk of clot:<br>Hold edoxaban. 24 hours after the last dose of edoxaban, start nirmatrelvir/ritonavir AND aspirin 81 mg daily. Finish aspirin 1 day after completing nirmaltrevir/ritonavir. Restart edoxaban 2 days after completing nirmatrelvir/ritonavir.<br>⚠ High risk of clot:<br>Hold edoxaban. 24 hours after the last dose of edoxaban, start nirmatrelvir/ritonavir AND therapeutic dosing of a subcutaneous low molecular weight heparin (LMWH) such as:<br>◦ **Dalteparin:** 200 units/kg daily OR 100 units/kg every 12 hours if >90 kg;<br>◦ **Enoxaparin:** 1 mg/kg every 12 hours (preferred) OR 1.5 mg/kg once every 24 hours;<br>◦ **Tinzaparin:** 175 anti-Xa units/kg once daily.<br>Finish LMWH 1 day after completing nirmatrelvir/ritonavir. Restart edoxaban 2 days after completing nirmatrelvir/ritonavir.<br>C) If atrial fibrillation:<br>Decrease edoxaban to 30 mg daily. Resume usual dose 2 days after completing nirmatrelvir/ritonavir.	No drug interaction data available with protease inhibitors but up to a 2-fold increase in exposure is anticipated.<br>Canadian product monograph recommends caution when using with ritonavir; 30 mg daily dose is recommended with P-glycoprotein inhibitors.<br>Lixiana (Canada). Product Monograph. Accessed December 6, 2023. https://pdf.hres.ca/dpd_pm/00063796.PDF<br>⚠ **High risk of clot includes:**<br>◦ Clot within past 6 months<br>◦ Clot at any time in past when anticoagulation interrupted<br>◦ Active cancer with clot at any point in cancer journey<br>◦ Diagnosis of antiphospholipid antibody syndrome<br>See *Paxlovid for a Patient on a DOAC* for more details. https://uwaterloo.ca/pharmacy/sites/default/files/uploads/documents/paxlovid_for_a_patient_on_a_doac_0.pdf
Elagolix (*Orilissa*)	Potential for increased elagolix concentrations and possibly decreased nirmatrelvir concentrations. Continue with usual elagolix dose during nirmatrelvir/ritonavir therapy and monitor for elagolix toxicity.	Potential for serious adverse effects, including suicidal ideation and elevation of hepatic transaminases.<br>Elagolix AUC increased over 2-fold when coadministered with ketoconazole 400 mg daily.
Elexacaftor/ tezacaftor/ ivacaftor (*Trikafta*)	**Reduce dose as follows:**<br>Day 1: Start nirmatrelvir/ritonavir. Take elexacaftor/tezacaftor/ivacaftor 200/100/150 mg (2 orange tablets) in the morning.<br>Day 2 to 4: No elexacaftor/tezacaftor/ivacaftor.<br>Day 5: Elexacaftor/tezacaftor/ivacaftor 200/100/150 mg (2 orange tablets) in the morning.<br>Day 6 to 8: No elexacaftor/tezacaftor/ivacaftor.<br>Day 9: Restart standard elexacaftor/tezacaftor/ivacaftor dose.<br>For patients where dose has previously been reduced for toxicity, consider alternative COVID-19 therapy.	Dosing recommendations based on a pharmacokinetic modelling study predicting a 9-fold increase in ivacaftor AUC, a 3-fold increase in tezacaftor AUC and a 2-fold increase in elexacaftor AUC following 5 days of coadministration with ritonavir.<br>Hong E, Almond LM, Chung PS et al. Physiologically-Based Pharmacokinetic-Led Guidance for Patients With Cystic Fibrosis Taking Elexacaftor-Tezacaftor-Ivacaftor With Nirmatrelvir-Ritonavir for the Treatment of COVID-19. *Clin Pharmacol Ther*. 2022;111(6):1324–1333. https://doi.org/10.1002/cpt.2585<br>Canadian product monograph recommends reducing the elexacaftor/tezacaftor/ivacaftordose dose to 200/100/150 mg twice weekly when coadministered with strong CYP3A4 inhibitors.<br>Trikafta (Canada). Product Monograph. Accessed December 6, 2023. https://pdf.hres.ca/dpd_pm/00061823.PDF
Encorafenib (*Braftovi*)	*Next page*	*Next page*

This document is intended for use by experienced clinicians, including prescribers and pharmacists. The information is not intended to replace sound professional judgment in individual situations, and should be used in conjunction with other reliable sources of information. Clinicians should always consider the risk/benefit profile for their individual patient, discuss these risks with the patient or caregiver before initiating therapy, and closely monitor for treatment benefit and adverse effects.

AUC = Area under the curve

Appendix (Page 7)

Drug	Recommendation	Comments
◆ Encorafenib (*Braftovi*)	Hold and restart 2 days after completing nirmatrelvir/ritonavir.<br>Alternatively, consider reducing encorafenib dose as follows and monitoring for toxicity:<br>• If taking 450 mg per day: reduce to 150 mg daily.<br>• If taking 150 to 300 mg per day: reduce dose to 75 mg daily.<br>Resume usual encorafenib dose 2 days after completing nirmatrelvir/ritonavir.	Decisions to hold or dose-adjust encorafenib should be made in conjunction with the patient's oncologist.<br>Encorafenib AUC increased 3-fold when coadministered with posaconazole.
● Eplerenone (*Inspra*)	Hold and restart 2 days after completing nirmatrelvir/ritonavir.<br>**If patient is at high risk and/or history of hyperkalemia:** Do not coadminister; use alternative COVID-19 therapy.	Co-administration with eplerenone is contraindicated due to potential for hyperkalemia.<br>Eplerenone AUC increased 441% when coadministered with ketoconazole.
● Ergot alkaloids (e.g., dihydroergotamine, ergonovine)	Hold and restart 2 days after completing nirmatrelvir/ritonavir.	Potential for serious and/or life threatening adverse effects, including acute ergot toxicity.
◆ Erythromycin	Use with caution and monitor for QTc prolongation in those at higher risk or consider using alternative antibiotic such as azithromycin.	
● Everolimus (*Certican*)	Decision to initiate nirmatrelvir/ritonavir should be done in conjunction with the patient's transplant provider.<br>Hold everolimus and start nirmatrelvir/ritonavir 12 hours after last everolimus dose.<br>Resuming transplant immunotherapy after the last dose of nirmatrelvir/ritonavir should be guided by therapeutic drug monitoring and in conjunction with the patient's transplant provider.	Check everolimus concentrations 2 days after the last dose of nirmatrelvir/ritonavir.<br>• If therapeutic/sub-therapeutic: Resume everolimus at 25 to 50% baseline dose. Repeat level every 2 to 4 days and adjust dose accordingly.<br>• If supratherapeutic: continue to hold everolimus; repeat level in 2 to 4 days to assess resumption.
◆ Felodipine	Reduce felodipine dose by 50% or take dose every other day. Restart usual dose 2 days after completing nirmatrelvir/ritonavir.<br>Monitor blood pressure. May consider continuing with usual dosing in patients at low risk of bradycardia or hypotension.	Concentrations of calcium channel blockers are expected to increase when coadministered with nirmatrelvir/ritonavir.
● Finerenone (*Kerendia*)	Hold and restart 2 days after completing nirmatrelvir/ritonavir.<br>**If patient is at high risk and/or history of hyperkalemia:** Do not coadminister; use alternative COVID-19 therapy.	Combination is contraindicated due to potential for serious adverse reactions including hyperkalemia, hypotension, and hyponatremia.
● Flibanserin (*Addyi*)	Ideally, hold flibanserin and start nirmatrelvir/ritonavir 2 days after the last dose of flibanserin. If this is not possible, hold flibanserin as soon as possible and monitor for hypotension and syncope. Restart flibanserin 2 weeks after completing nirmatrelvir/ritonavir.	Flibanserin AUC increased AUC 4.5-fold when coadministered with ketoconazole. Coadministration of flibanserin is contraindicated with moderate or strong CYP3A4 inhibitors.
● Flurazepam	Hold and restart 2 days after completing nirmatrelvir/ritonavir.<br>If an anxiolytic is needed, use lorazepam, oxazepam, or temazepam at usual doses.	Due to prolonged benzodiazepine half-life, coadministration is not recommended.
◆ Fostamatinib (*Tavalisse*)	Monitor for toxicity including diarrhea, hypertension, hepatotoxicity, and neutropenia. If significant toxicity occurs, consider interruption of fostamatinib with reintroduction 2 days after completing nirmatrelvir/ritonavir.	Fostamatinib active metabolite AUC increased 102% when coadministered with ketoconazole.

This document is intended for use by experienced clinicians, including prescribers and pharmacists. The information is not intended to replace sound professional judgment in individual situations, and should be used in conjunction with other reliable sources of information. Clinicians should always consider the risk/benefit profile for their individual patient, discuss these risks with the patient or caregiver before initiating therapy, and closely monitor for treatment benefit and adverse effects.

AUC = Area under the curve

Appendix (Page 8)

Drug	Recommendation	Comments
● Glecaprevir/ Pibrentasvir (*Maviret*)	If possible, use alternative COVID-19 agent or consult a Hepatitis C (HCV) specialist. If under specialist guidance, may coadminister with caution and monitor for liver toxicity.	Glecaprevir exposure is increased over 4-fold with ritonavir and is associated with increased risk of alanine aminotransferase (ALT) elevation.<br><br>In patients who are planning to start Hepatitis C (HCV) treatment, glecaprevir/pibrentasvir treatment should be deferred.
◆ Hydrocodone	Reduce dose by about 50% or switch to equivalent dose of hydromorphone:<br>• Multiply hydrocodone dose by 0.25 to get equivalent hydromorphone dose.<br>• Consider further reducing hydromorphone dose by 25 to 50% to account for cross tolerance.<br><br>Monitor for signs of opioid toxicity. Resume usual hydrocodone dose 2 days after completing nirmatrelvir/ritonavir.	Hydrocodone is metabolized to active metabolites: hydromorphone and norhydrocodone.<br><br>Hydrocodone AUC increased by 90% when coadministered with ritonavir/ombitasvir/ paritaprevir combination.
● Ibrutinib (*Imbruvica*)	Consider alternate COVID-19 therapy.<br><br>Alternatively, consider holding ibrutinib and starting nirmatrelvir/ritonavir 12 hours after the last ibrutinib dose. Restart ibrutinib 2 days after completing nirmatrelvir/ritonavir.	Decisions to hold or dose-adjust ibrutinib should be made in conjunction with the patient's oncologist. **It may be dangerous to interrupt therapy in patients with high volume chronic lymphocytic leukemia or mantle cell lymphoma due to disease flare and/or cytokine release.**<br><br>Ibrutinib AUC increased 26-fold when coadministered with ketoconazole.
◆ Isavuconazole (*Cresemba*)	Monitoring is advised based on potential risk of increased side effects of isavuconazole.	Contraindicated with ketoconazole.<br><br>For the strong CYP3A4 inhibitor lopinavir/ritonavir, a 2-fold increase (96% incr AUC) in isavuconazole exposure was observed. For other strong CYP3A4 inhibitors, such as clarithromycin, indinavir, and saquinavir, a less pronounced effect can be expected based on their relative potency. No dose adjustment of isavuconazole is necessary when co-administered with these strong CYP3A4/5 inhibitors. However, caution is advised as adverse drug reactions may increase.<br>Cresemba (Canada). Product Monograph. Accessed December 6, 2023. https://pdf.hres.ca/dpd_pm/00048808.PDF
◆ Ivacaftor (*Kalydeco*)	**Reduce dose as follows:**<br>Day 1: Start nirmatrelvir/ritonavir. Take ivacaftor 150 mg in the morning only.<br>Day 2 to 4: No ivacaftor.<br>Day 5: Take ivacaftor 150 mg in the morning only.<br>Day 6 to 8: No ivacaftor.<br>Day 9: Restart standard ivacaftor dose.<br><br>For patients where dose has previously been reduced for toxicity, consider alternative COVID-19 therapy.	Dosing recommendations based on a pharmacokinetic modelling study predicting a 9-fold increase in ivacaftor AUC following 5 days of coadministration with ritonavir.<br>Hong E, Almond LM, Chung PS et al. Physiologically-Based Pharmacokinetic-Led Guidance for Patients With Cystic Fibrosis Taking Elexacaftor-Tezacaftor-Ivacaftor With Nirmatrelvir-Ritonavir for the Treatment of COVID-19. *Clin Pharmacol Ther*. 2022;111(6):1324–1333. https://doi.org/10.1002/cpt.2585<br><br>Canadian product monograph recommends reducing the ivacaftor dose to 150 mg twice weekly when coadministered with strong CYP3A4 inhibitors.<br>Kalydeco (Canada). Product Monograph. Accessed December 6, 2023. https://pdf.hres.ca/dpd_pm/00042032.PDF
● Lomitapide (*Juxtapid*)	Hold and restart 2 days after completing nirmatrelvir/ritonavir.	Lomitapide AUC increased 27-fold when coadministered with ketoconazole.
● Lovastatin	Stop lovastatin at least 12 hours before starting nirmatrelvir/ritonavir. Restart 5 days after completing nirmatrelvir/ritonavir.	Contraindicated due to potential for severe toxicity including rhabdomyolysis and elevated liver function tests.

This document is intended for use by experienced clinicians, including prescribers and pharmacists. The information is not intended to replace sound professional judgment in individual situations, and should be used in conjunction with other reliable sources of information. Clinicians should always consider the risk/benefit profile for their individual patient, discuss these risks with the patient or caregiver before initiating therapy, and closely monitor for treatment benefit and adverse effects.

AUC = Area under the curve

Appendix (Page 9)

Drug	Recommendation	Comments
● Meperidine (*Demerol*)	Do not coadminister. Switch meperidine to an equivalent dose of hydromorphone:<br>• Multiply meperidine dose by 0.02 to get equivalent hydromorphone dose.<br>• Consider further reducing hydromorphone dose by 25 to 50% to account for cross tolerance.<br><br>Monitor for signs of opioid toxicity. Resume usual meperidine dose 2 days after completing nirmatrelvir/ritonavir.	Normeperidine AUC increased 50% when coadministered with ritonavir.<br><br>Higher levels of normeperidine can cause central nervous system excitation and seizures.
● Midazolam, oral	Hold and restart 2 days after completing nirmatrelvir/ritonavir.	Combination is contraindicated.<br>Coadministration may result in large increases in oral midazolam concentrations with the potential for serious events such as prolonged or increased sedation or respiratory depression.
◆ Modafinil	No dose adjustment required. Monitor for anxiety and agitation.	Coadministration could potentially increase modafinil exposure due to CYP3A4 inhibition. Modafinil is a moderate inducer of CYP3A4, but a clinically significant effect on nirmetrelvir/ritonavir exposure is unlikely.
● Naloxegol (*Movantik*)	Coadministration is contraindicated due to the potential for adverse effects including abdominal pain, diarrhea, nausea, flatulence, vomiting, headache, and hyperhidrosis and withdrawal symptoms. Hold naloxegol and restart at least 2 days after completing nirmatrelvir/ritonavir.	Naloxegol AUC increased almost 13-fold when coadministered with ketoconazole.
● Neratinib (*Nerlynx*)	Hold and start nirmatrelvir/ritonavir 24 hours after the last neratinib dose. Restart neratinib 2 days after completing nirmatrelvir/ritonavir.	Decisions to hold or dose-adjust should be made in conjunction with the patient's oncologist.<br><br>Neratinib AUC increased almost 5-fold when coadministered with ketoconazole.
◆ Nifedipine	Reduce nifedipine dose by 50% or take dose every other day. Restart usual dose 2 days after completing nirmatrelvir/ritonavir.<br><br>Monitor blood pressure. May consider continuing with usual dosing in patients at low risk of bradycardia or hypotension.	Concentrations of calcium channel blockers are expected to increase when coadministered with nirmatrelvir/ritonavir.
◆ Nilotinib (*Tasigna*)	**Chronic phase chronic myelogenous leukemia (CML):**<br>Hold nilotinib if possible, restart 2 days after completing nirmatrelvir/ritonavir. Alternatively, consider dose reduction to 400 mg PO daily and monitor for toxicity.<br><br>**Accelerated or blast phase CML:**<br>Do not coadminister. Consider an alternate COVID-19 therapy.	Decisions to hold or dose-adjust nilotinib should be made in conjunction with the patient's oncologist.<br><br>Canadian monograph recommends holding if using CYP3A4 inhibitors, or monitoring for QTc if treatment interruption is not possible. A 50% dose reduction is recommended based on expected effect on nilotinib exposures.<br>Deeken JF, Pantanowitz I, Dezube BJ. Targeted therapies to treat non-AIDS-defining cancers in patients with HIV on HAART therapy: treatment considerations. *Curr Opin Oncol* 2009; 21(5): 445-54. https://doi.org/10.1097/cco.0b013e32832f3e04<br><br>Nilotinib AUC increased 3-fold when coadministered with ketoconazole.
● Nitrazepam (*Mogadon*)	Hold and restart 2 days after completing nirmatrelvir/ritonavir.<br><br>If an anxiolytic is needed, use lorazepam, oxazepam, or temazepam at usual doses.	Due to prolonged benzodiazepine half-life, coadministration is not recommended.
◆ Oxycodone (*Percocet, OxyNEO*)	*Next page*	*Next page*

This document is intended for use by experienced clinicians, including prescribers and pharmacists. The information is not intended to replace sound professional judgment in individual situations, and should be used in conjunction with other reliable sources of information. Clinicians should always consider the risk/benefit profile for their individual patient, discuss these risks with the patient or caregiver before initiating therapy, and closely monitor for treatment benefit and adverse effects.

AUC = Area under the curve

Appendix (Page 10)

Drug	Recommendation	Comments
◆ Oxycodone (*Percocet, OxyNEO*)	Reduce dose of oxycodone by 66% or switch to equivalent dose of hydromorphone:<br>• Multiply oxycodone dose by 0.3 to get equivalent hydromorphone dose.<br>• Consider further reducing hydromorphone dose by 25 to 50% to account for cross tolerance.<br><br>Monitor for signs of opioid toxicity. Resume usual oxycodone dose 2 days after completing nirmatrelvir/ritonavir.	Oxycodone half-life increased 2-fold and AUC increased between 3 and 4-fold when coadministered with other potent 3A4 inhibitors (i.e., voriconazole).
◆ Quetiapine (*Seroquel*)	Reduce to one-sixth of original dose and resume usual dose 2 days after completing nirmatrelvir/ritonavir.<br><br>Monitor for confusion, dizziness, and sedation.	Quetiapine AUC increased 5 to 8-fold when coadministered with ketoconazole.
● Quinine	**For treatment of leg cramps:**<br>Hold and restart 2 days after completing nirmatrelvir/ritonavir.<br><br>**For treatment of malaria:**<br>Use an alternative COVID-19 agent.	Quinine AUC increased 4-fold and conversion to active metabolite was markedly inhibited when coadministered with ritonavir 200 mg twice daily.
◆ Rifabutin	Reduce rifabutin to 150 mg once daily; return to 300 mg once daily 2 days after completing nirmatrelvir/ritonavir.	Canadian monograph recommends 150 mg three times a week, but the dose has been found to be too low and contributes to resistance. The Department of Health and Human Services Panel on Antiretroviral Guidelines for Adults and Adolescents recommends using rifabutin 150 mg daily when used with a ritonavir-boosted protease inhibitor.<br>https://clinicalinfo.hiv.gov/en/guidelines/adult-and-adolescent-arv/drug-interactions-between-protease-inhibitors-and-other-drugs?view=full<br><br>Significant increases in exposures of rifabutin (>3-fold) and metabolite (>40-fold) observed when coadministered with lopinavir/ritonavir 400/100 mg twice daily.
◆ Risperidone (*Risperdal*), oral	Reduce risperidone dose by 25 to 50% and resume usual dose 2 days after completing nirmatrelvir/ritonavir.<br><br>Monitor for confusion, extrapyramidal symptoms, and sedation.	Risperidone AUC increased up to 2-fold when coadministered with ketoconazole.<br><br>Avoid coadministration in patients stabilized on risperidone long-acting injection.
● Rivaroxaban (*Xarelto*)	*Next page*	*Next page*

This document is intended for use by experienced clinicians, including prescribers and pharmacists. The information is not intended to replace sound professional judgment in individual situations, and should be used in conjunction with other reliable sources of information. Clinicians should always consider the risk/benefit profile for their individual patient, discuss these risks with the patient or caregiver before initiating therapy, and closely monitor for treatment benefit and adverse effects.

TIA = Transient ischemic attack
AUC = Area under the curve

Appendix (Page 11)

Drug	Recommendation	Comments
● Rivaroxaban (*Xarelto*)	**If possible, use alternative COVID-19 agent. If not possible, then:**<br>A) If acute venous thromboembolism (VTE):<br>⚠ Low risk of clot:<br>Hold rivaroxaban. 24 hours after the last dose of rivaroxaban, start nirmatrelvir/ritonavir AND aspirin 81 mg daily. Finish aspirin 1 day after completing nirmaltrevir/ ritonavir. Restart rivaroxaban 2 days after completing nirmatrelvir/ritonavir.<br>⚠ High risk of clot:<br>Hold rivaroxaban. 24 hours after the last dose of rivaroxaban, start nirmatrelvir/ritonavir AND therapeutic dosing of a subcutaneous low molecular weight heparin (LMWH) such as:<br>◦ **Dalteparin:** 200 units/kg daily OR 100 units/kg every 12 hours if >90 kg;<br>◦ **Enoxaparin:** 1 mg/kg every 12 hours (preferred) OR 1.5 mg/kg once every 24 hours;<br>◦ **Tinzaparin:** 175 anti-Xa units/kg once daily.<br>Finish LMWH 1 day after completing nirmatrelvir/ritonavir. Restart rivaroxaban 2 days after completing nirmatrelvir/ ritonavir.<br>B) If atrial fibrillation:<br>Hold rivaroxaban. 24 hours after the last dose of rivaroxaban, start nirmatrelvir/ritonavir AND edoxaban 30 mg daily. Finish edoxaban 1 day after completing nirmatrelvir/ritonavir. Restart rivaroxaban 2 days after completing nirmatrelvir/ ritonavir.<br>For patients currently receiving rivaroxaban 20 mg daily, if <65 years old with CrCl >50 mL/min:<br>It may be reasonable to decrease rivaroxaban to 10 mg daily. Resume normal rivaroxaban dose 2 days after completing nirmatrelvir/ritonavir.	Rivaroxaban AUC and Cmax increased by 153% and 55%, respectively, when coadministered with ritonavir 600 mg twice daily in healthy volunteers.<br>⚠ **High risk of clot includes:**<br>◦ Clot within past 6 months<br>◦ Clot at any time in past when anticoagulation interrupted<br>◦ Active cancer with clot at any point in cancer journey<br>◦ Diagnosis of antiphospholipid antibody syndrome<br>See *Paxlovid for a Patient on a DOAC* for more details. https://uwaterloo.ca/pharmacy/sites/default/files/uploads/documents/paxlovid_for_a_patient_on_a_doac_0.pdf<br>A pharmacokinetic modelling study suggested that decreasing rivaroxaban to 10 mg daily during nirmatrelvir/ritonavir therapy maintained acceptable rivaroxaban exposures in the general population with no increased risk of major bleeding. However, the risk of major bleeding was increased in the geriatric population and those with impaired renal function.<br>Wang Z, Chan ECY. Physiologically-based pharmacokinetic modeling-guided dose management of oral anticoagulants when initiating nirmatrelvir/ritonavir (Paxlovid) for COVID-19 treatment. *Clin Pharmacol Ther* 2022;112:803-807. https://doi.org/10.1002/cpt.2687<br>Observational data from the US found no increased risk of bleeding in those prescribed apixaban, rivaroxaban, and dabigatran with nirmatrelvir/ritonavir, even when recommended dosing adjustments were not followed.<br>Vazquez S, Wilson A, Witt D. Management of potential drug-drug interactions with nirmatrelvir-ritonavir and oral anticoagulants: a case series. *J Thromb Thrombolysis*. 2022;54:583–86. https://www.ncbi.nlm.nih.gov/pmc/articles/PMC9579540/
◆ Rosuvastatin	Hold and restart 2 days after completing nirmatrelvir/ritonavir.<br>Alternatively, reduce rosuvastatin to the equivalent of 10 mg daily by alternative day dosing, cutting the tablet, or obtaining a new prescription. Resume usual dose 2 days after completing nirmatrelvir/ritonavir.	Rosuvastatin AUC increased 2-fold and Cmax increased almost 5-fold when coadministered with lopinavir/ritonavir 400/100 mg twice daily.
● Salmeterol (*Serevent, Advair*)	Hold and restart 2 days after completing nirmatrelvir/ritonavir.	Potential for serious and/or life-threatening adverse effects, including cardiac arrhythmias (prolonged QTc).
◆ Saxagliptin (*Onglyza*)	Reduce saxagliptin to 2.5 mg once daily and resume usual dose 2 days after completing nirmatrelvir/ritonavir.	Saxagliptin AUC increased 145% when coadministered with ketoconazole.
◆ Sildenafil for erectile dysfunction (*Viagra*)	Hold and restart 2 days after completing nirmatrelvir/ritonavir.<br>Alternatively, reduce dose to 25 mg once every 48 hours. Resume usual dose 2 days after completing nirmatrelvir/ritonavir.	Sildenafil AUC increased 2 to 11-fold when coadministered with protease inhibitors.

This document is intended for use by experienced clinicians, including prescribers and pharmacists. The information is not intended to replace sound professional judgment in individual situations, and should be used in conjunction with other reliable sources of information. Clinicians should always consider the risk/benefit profile for their individual patient, discuss these risks with the patient or caregiver before initiating therapy, and closely monitor for treatment benefit and adverse effects.

Cmax = Maximum (or peak) serum concentration
AUC = Area under the curve

Appendix (Page 12)

Drug	Recommendation	Comments
● Silodosin (*Rapaflo*)	Hold and restart 2 days after completing nirmatrelvir/ritonavir.	Silodosin AUC increased over 3-fold when coadministered with ketoconazole.
● Simvastatin	Stop simvastatin at least 12 hours before starting nirmatrelvir/ritonavir. Restart 5 days after completing nirmatrelvir/ritonavir.	Contraindicated due to potential for severe toxicity including rhabdomyolysis and elevated liver function tests.
● Sirolimus (*Rapamune*)	Decision to initiate nirmatrelvir/ritonavir should be done in conjunction with the patient's transplant provider.<br><br>Hold sirolimus and start nirmatrelvir/ritonavir 24 to 48 hours after the last sirolimus dose.<br><br>Resuming transplant immunotherapy after the last dose of nirmatrelvir/ritonavir should be guided by therapeutic drug monitoring and in conjunction with the patient's transplant provider.	Check sirolimus concentration 2 days after the last dose of nirmatrelvir/ritonavir.<br>• If therapeutic/subtherapeutic: Resume sirolimus at 50% of baseline dose. Repeat level every 7 days and dose-adjust accordingly.<br>• If supratherapeutic: Continue to hold sirolimus and repeat level in 5 to 7 days to assess resumption.
● Tacrolimus (*Prograf, Advagraf, Envarsus*)	Decision to initiate nirmatrelvir/ritonavir should be done in conjunction with the patient's transplant provider.<br><br><u>Immediate release (*Prograf*, generics):</u><br>Hold tacrolimus and start nirmatrelvir/ritonavir 12 hours after the last tacrolimus dose.<br><br><u>Extended (*Advagraf*) or prolonged (*Envarsus*) release:</u><br>Hold the long acting tacrolimus and start nirmatrelvir/ritonavir 24 hours after the last tacrolimus dose.<br><br>Resuming transplant immunotherapy after the last dose of nirmatrelvir/ritonavir should be guided by therapeutic drug monitoring and in conjunction with the patient's transplant provider.	For all forms of tacrolimus: check tacrolimus concentrations 2 days after the last dose of nirmatrelvir/ritonavir.<br>• If subtherapeutic: Resume tacrolimus at 75% of baseline dose for 3 days, then resume baseline dose (100% of original dose). Repeat level 1 week later.<br>• If therapeutic: Resume tacrolimus at 50% of baseline dose for 3 days, then resume baseline dose (100% of original dose). Repeat level 1 week later.<br>• If supratherapeutic: Resume tacrolimus at 33% of baseline dose for 3 days, then repeat level in 2 to 4 days to guide further dosing.<br><br>Giguère P, Deschenes MJ, Van Loon M et al. Management and outcome of COVID-19 infection using nirmaltrevir/ritonavir in kidney transplant patients. *Clin J Am Soc Nephron*. 2023;18:913-919. https://doi.org/10.2215/cjn.0000000000000186
◆ Tadalafil for erectile dysfunction (*Cialis*)	Hold and restart 2 days after completing nirmatrelvir/ritonavir.<br><br>Alternatively, reduce the dose to 10 mg once every 72 hours. Resume usual dose 2 days after completing nirmatrelvir/ritonavir.	Tadalafil AUC increased 124% when coadministered with ritonavir 200 mg twice daily.
◆ Tamsulosin (*Flomax*)	Hold and restart 2 days after completing nirmatrelvir/ritonavir. Alternatively, may consider using 0.4 mg daily or giving every other day in patients with heightened risk of urinary retention. Monitor for hypotension. Resume usual dose 2 days after completing nirmatrelvir/ritonavir.	Tamsulosin AUC increased almost 3-fold when coadministered with ketoconazole.
◆ Tezacaftor/ ivacaftor (*Symdeko*)	*Next page*	*Next page*

This document is intended for use by experienced clinicians, including prescribers and pharmacists. The information is not intended to replace sound professional judgment in individual situations, and should be used in conjunction with other reliable sources of information. Clinicians should always consider the risk/benefit profile for their individual patient, discuss these risks with the patient or caregiver before initiating therapy, and closely monitor for treatment benefit and adverse effects.

AUC = Area under the curve

Appendix (Page 13)

Drug	Recommendation	Comments
◆ Tezacaftor/ ivacaftor (*Symdeko*)	**Reduce dose as follows:**<br>Day 1: Start nirmatrelvir/ritonavir. Take tezacaftor/ivacaftor 100/150 mg (1 yellow tablet) in the morning.<br>Day 2 to 4: No tezacaftor/ivacaftor.<br>Day 5: Tezacaftor/ivacaftor 100/150 mg (1 yellow tablet) in the morning.<br>Day 6 to 8: No tezacaftor/ivacaftor.<br>Day 9: Restart standard tezacaftor/ivacaftor dose.<br><br>For patients where dose has previously been reduced for toxicity, consider alternative COVID-19 therapy.	Dosing recommendations based on a pharmacokinetic modelling study predicting a 9-fold increase in ivacaftor AUC and a 3-fold increase in tezacaftor AUC following 5 days of coadministration with ritonavir.<br>Hong E, Almond LM, Chung PS et al. Physiologically-Based Pharmacokinetic-Led Guidance for Patients With Cystic Fibrosis Taking Elexacaftor-Tezacaftor-Ivacaftor With Nirmatrelvir-Ritonavir for the Treatment of COVID-19. *Clin Pharmacol Ther*. 2022;111(6):1324–1333. https://doi.org/10.1002/cpt.2585<br><br>Canadian product monograph recommends reducing tezacaftor/ivacaftor dose to 100/150 mg twice weekly when coadministered with strong CYP3A4 inhibitors.<br>Symdeko (Canada). Product Monograph. Accessed December 6, 2023. https://pdf.hres.ca/dpd_pm/00046145.PDF
● Ticagrelor (*Brilinta*)	**Acute coronary syndrome (ACS)/percutaneous coronary intervention (PCI):**<br>• If <1 month since ACS: Suggest alternative COVID-19 agent.<br>• If <3 months since ACS or <1 month since PCI (no ACS): Switch to prasugrel (if age <75, weight >60 kg, and no history of stroke/TIA) during nirmatrelvir/ritonavir therapy.<br>• If >3 months since ACS or >1 month since PCI (no ACS): Consider temporarily holding ticagrelor (i.e., no switching) during nirmatrelvir/ritonavir therapy and resuming after. If not taking acetylsalicylic acid (ASA), consider switching to prasugrel (if age <70, weight >60 kg, and no history of stroke/TIA) or half-dose of ticagrelor (45 mg twice daily).	Ticagrelor AUC increased 36% when coadministered with a single dose of ritonavir 100 mg.
◆ Tofacitinib (*Xeljanz*)	Hold and restart 2 days after completing nirmaltrelvir/ritonavir. Alternatively, reduce tofacitinib dose by 50% or take dose every other day. Restart usual dose 2 days after completing nirmatrelvir/ritonavir.<br><br>It is recommended to hold tofacitinib in the case of serious infections.	Tofacitinib AUC increased 2-fold when coadministered with ketoconazole.
◆ Tramadol	Reduce tramadol dose by 50% and monitor for pain relief and opioid toxicity. Resume usual dose 2 days after completing nirmatrelvir/ritonavir.	Inhibition of CYP3A4 may increase tramadol concentrations. Inhibition of CYP2D6 can decrease conversion of tramadol to a more active metabolite, but this is not expected to be significant when coadministered with nirmatrelvir/ritonavir.
◆ Trazodone (*Desyrel*)	*Next page*	*Next page*

This document is intended for use by experienced clinicians, including prescribers and pharmacists. The information is not intended to replace sound professional judgment in individual situations, and should be used in conjunction with other reliable sources of information. Clinicians should always consider the risk/benefit profile for their individual patient, discuss these risks with the patient or caregiver before initiating therapy, and closely monitor for treatment benefit and adverse effects.

AUC = Area under the curve

Appendix (Page 14)

Drug	Recommendation	Comments
◆ Trazodone (*Desyrel*)	Reduce trazodone dose by 50%. Restart usual dose 2 days after completing nirmatrelvir/ritonavir.	Trazodone AUC increased over 2-fold when coadministered with ritonavir 200 mg twice daily.
● Triazolam (*Halcion*)	Hold and restart 2 days after completing nirmatrelvir/ritonavir.<br>If an anxiolytic is needed, use lorazepam, oxazepam, or temazepam at usual doses.	Combination is contraindicated. Coadministration may result in large increases in triazolam concentrations with the potential for serious events such as prolonged or increased sedation or respiratory depression.
● Ubrogepant (*Ubrelvy*)	Stop ubrogepant at least 12 hours before starting nirmaltrevir/ritonavir. Restart ubrogepant 2 days after completing nirmatrelvir/ritonavir.	Combination is contraindicated. Ubrogepant AUC increased 10-fold when coadministered with ketoconazole.
◆ Upadacitinib (*Rinvoq*)	If on 15 mg daily, continue upadacitinib usual dosing. If taking upadacitinib 30 mg daily, consider dosing every other day. If taking 45 mg daily, consider reduction to 30 mg daily. Restart previous dose 2 days after completion of nirmatrelvir/ritonavir.<br>It is recommended to hold upadactinib in the case of severe infections.	Upadacitinib AUC increased 75% when coadministered with ketoconazole.
● Vardenafil (*Levitra*) for erectile dysfunction	Hold and restart 2 days after completing nirmatrelvir/ritonavir.	Vardenafil AUC increased 49-fold when coadministered with ritonavir 600 mg twice daily.
◆ Verapamil	Reduce verapamil dose by 50% or take dose every other day. Restart usual dose 2 days after completing nirmatrelvir/ritonavir.<br>Monitor blood pressure. May consider continuing with usual dosing in patients at low risk of bradycardia or hypotension.	Concentrations of calcium channel blockers are expected to increase when coadministered with nirmatrelvir/ritonavir.
◆ Vinblastine	Vinblastine may be held in the context of acute infection. Restart vinblastine at least 2 days after completing nirmatrelvir/ritonavir.<br>Alternatively, vinblastine may be coadministered with close monitoring for hematologic and neurotoxicity. Some providers may wish to empirically reduce vinblastine dose, especially in patients who have previously experienced or are at high risk for toxicity.	Decisions to hold or dose-adjust should be made in conjunction with the patient's oncologist.<br>Vinblastine AUC increased almost 2-fold when coadministered with ritonavir. Increased risk of autonomic and peripheral neurotoxicity and neutropenia have been reported with coadministration of ritonavir and vinblastine.
◆ Vincristine	Vincristine may be held in the context of acute infection. Restart vincristine 2 days after completing nirmatrelvir/ritonavir.<br>Alternatively, vincristine may be coadministered with close monitoring for hematologic and neurotoxicity. Some providers may wish to empirically reduce vincristine dose, especially in patients who have previously experienced or are at high risk for toxicity.	Decisions to hold or dose-adjust should be made in conjunction with the patient's oncologist.<br>Increased rates of hematologic toxicity and neuropathy (including autonomic neuropathy) have been reported with coadministration of ritonavir and vincristine.
◆ Voriconazole (*Vfend*)	*Next page*	*Next page*

This document is intended for use by experienced clinicians, including prescribers and pharmacists. The information is not intended to replace sound professional judgment in individual situations, and should be used in conjunction with other reliable sources of information. Clinicians should always consider the risk/benefit profile for their individual patient, discuss these risks with the patient or caregiver before initiating therapy, and closely monitor for treatment benefit and adverse effects.

AUC = Area under the curve

Appendix (Page 15)

Drug	Recommendation	Comments
Voriconazole (*Vfend*)	Risks and benefits should be evaluated to either hold or continue voriconazole during treatment with nirmatrelvir/ritonavir.	Co-administration with nirmatrelvir/ritonavir has led to an increase or decrease in voriconazole exposure, based mainly on CYP2C19 metabolizer status. Normal CYP2C19 metabolizers experienced reduced voriconazole levels while intermediate and poor metabolizers had an increase in concentration.<br>Wang P, Xing H, Zhang X, Yang J. Complexity interactions between nirmatrelvir/ritonavir and voriconazole in patients with coronavirus disease 2019. *Clin Infect Dis*. 2023; 76(12):2209-2210. https://doi.org/10.1093/cid/ciad159<br>Bayo EH, Andreu MM, Lacunza RH et al. [Translated article] Paradoxical interaction between nirmatrelvir/ritonavir and voriconazole in a patient with COVID-19. *Farm Hosp*. 2023; 47(2):T93-T95. https://doi.org/10.1016/j.farma.2023.02.003
Warfarin	Monitor for signs of increased bleeding and bruising. Check international normalized ratio (INR) if clinically indicated.	Potential for increased warfarin concentrations when coadministered with nirmatrelvir/ritonavir.
Ziprasidone (*Zeldox*)	No dose adjustment required. Monitor for dizziness, extrapyramidal symptoms, and sedation.	Only one-third of ziprasidone dose is metabolized by CYP450. Ziprasidone AUC increased 35 to 40% when coadministered with ketoconazole.
Zolpidem (*Sublinox, Ambien*)	Hold and restart 2 days after completing nirmatrelvir/ritonavir.<br>If coadministration required, reduce zolpidem dose by 50%.	Zolpidem AUC increased 70% when coadministered with ketoconazole.
Zopiclone (*Imovane*)	Hold and restart 2 days after completing nirmatrelvir/ritonavir.<br>If coadministration required, reduce zopiclone dose by 50%.	Potential for increased zopiclone exposures when coadministered with nirmatrelvir/ritonavir.

This document is intended for use by experienced clinicians, including prescribers and pharmacists. The information is not intended to replace sound professional judgment in individual situations, and should be used in conjunction with other reliable sources of information. Clinicians should always consider the risk/benefit profile for their individual patient, discuss these risks with the patient or caregiver before initiating therapy, and closely monitor for treatment benefit and adverse effects.

AUC = Area under the curve

UNIVERSITY OF WATERLOO | SCHOOL OF PHARMACY

UNIVERSITY OF TORONTO
LESLIE DAN FACULTY OF PHARMACY

Updated: December 12, 2022

PAXLOVID™
FOR A PATIENT ON A DOAC (DIRECT ORAL ANTICOAGULANT)

who is also at high risk of hospitalization from COVID-19
(e.g., unvaccinated, immunocompromised, or over 60 years old)

RIVAROXABAN

Is remdesivir an option?
- YES → **REFER PATIENT FOR REMDESIVIR**
- NO → **WHY IS THE PATIENT ON RIVAROXABAN?**
  - → ATRIAL FIBRILLATION
  - → VTE/ATE

APIXABAN, DABIGATRAN, EDOXABAN

Is remdesivir an option?
- YES → **REFER PATIENT FOR REMDESIVIR**
- NO → **IS THE PATIENT ON A LOW DOSE?**

APIXABAN 2.5 MG BID
DABIGATRAN 110 MG BID
EDOXABAN ≤ 30 MG DAILY

- YES → **CONTINUE WITHOUT CHANGE***
- NO → **WHY IS THE PATIENT ON APIXABAN/DABIGATRAN/EDOXABAN?**
  - → VTE/ATE
  - → ATRIAL FIBRILLATION

ATRIAL FIBRILLATION
+/- bioprosthetic valve

Hold rivaroxaban, start edoxaban* *(30 mg daily)*

Page 2

For patients currently receiving rivaroxaban 20 mg daily, if <65 years old with CrCl>50 mL/min, it may be reasonable to decrease rivaroxaban to 10 mg daily. Resume normal rivaroxaban dose 2 days after completing nirmatrelvir/ritonavir.†

†Wang Z, Chan ECY. *Clin Pharmacol Ther* 2022;112:803-807.

VTE/ATE
Venous thromboembolism/arterial thromboembolism

LOW RISK OF CLOT

Hold DOAC, start aspirin*

Page 2

HIGH RISK OF CLOT

Hold DOAC, start LMWH*

Page 2

LMWH = Low molecular weight heparin

High risk of clot includes:
- Clot within past 6 months
- Clot at any time in past when anticoagulation interrupted
- Active cancer with clot at any point in cancer journey
- Diagnosis of antiphospholipid antibody syndrome

ATRIAL FIBRILLATION
+/- bioprosthetic valve

CHANGE TO LOW DOSE

Apixaban	Reduce to 2.5 mg BID
Edoxaban	Reduce to 30 mg daily
Dabigatran	Reduce to 110 mg BID *(eGFR >50 mL/minute)*<br>Reduce to 75 mg BID *(eGFR 30-50 mL/minute)*

Resume usual dose 2 days after completing Paxlovid™.

***Decisions to hold, adjust, or change medications should be made on a patient-specific basis.**

This document is intended for use by experienced clinicians, including prescribers and pharmacists. The information is not intended to replace sound professional judgment in individual situations, and should be used in conjunction with other reliable sources of information. Clinicians should always consider the risk/benefit profile for their individual patient, discuss these risks with the patient or caregiver before initiating therapy, and closely monitor for treatment benefit and adverse effects.

How to hold a DOAC and start aspirin:

VTE/ATE: LOW RISK OF CLOT

DAY 0
Last dose of DOAC

apixaban OR *dabigatran* **wait 12h**
edoxaban OR *rivaroxaban* **wait 24h** *

DAY 1
Start Paxlovid™ AND **aspirin** *(81 mg daily)*

DAY 5
Finish Paxlovid™

DAY 6
Last dose of aspirin

DAY 7†
Restart DOAC

wait at least 24h

**Interval may be shortened if patient is at end of Paxlovid™ eligibility time window.*

† If Paxlovid is started in evening of Day 1 and last dose is morning of Day 6, last dose of aspirin will be Day 7, and restarting DOAC will be Day 8.

How to hold a DOAC and start LMWH:

VTE/ATE: HIGH RISK OF CLOT

DAY 0
Last dose of DOAC

apixaban OR *dabigatran* **wait 12h**
edoxaban OR *rivaroxaban* **wait 24h** *

DAY 1
Start Paxlovid™ AND **LMWH**

DAY 5
Finish Paxlovid™

DAY 6
Last dose of LMWH

DAY 7†
Restart DOAC

wait at least 24h

**Interval may be shortened if patient is at end of Paxlovid™ eligibility time window.*

**Possible LMWH doses:**	***Dalteparin*** 200 units/kg daily *If >90 kg:* 100 units/kg (every 12h)	***Enoxaparin*** 1 mg/kg every 12h *(preferred)* OR 1.5 mg/kg once every 24h	***Tinzaparin*** 175 anti-Xa units/kg once daily

† If Paxlovid is started in evening of Day 1 and last dose is morning of Day 6, last dose of LMWH will be Day 7, and restarting DOAC will be Day 8.

How to hold rivaroxaban and start edoxaban:

ATRIAL FIBRILLATION

DAY 0
Last dose of rivaroxaban

wait 24h *

DAY 1
Start Paxlovid™ AND **edoxaban** *(30 mg daily)*

DAY 5
Finish Paxlovid™

DAY 6
Last dose of edoxaban

DAY 7†
Restart rivaroxaban

wait at least 24h

**Interval may be shortened if patient is at end of Paxlovid™ eligibility time window.*

† If Paxlovid is started in evening of Day 1 and last dose is morning of Day 6, last dose of edoxaban will be Day 7, and restarting DOAC will be Day 8.

People who take a DOAC should stay up to date with their COVID-19 vaccines, including boosters.

It can be challenging to manage drug interactions between COVID-19 treatments and DOACs. Vaccination can reduce the risk of needing treatment for COVID-19.

Contributors: Kelly Grindrod, BScPharm, PharmD, MSc. Claudia Bucci, BScPhm, PharmD, ACPR. Stephanie Carlin, PharmD, ACPR2. Andrea Chittle, MD, CCFP. Noah Ivers, MD, PhD, CCFP. Heather Kertland, PharmD, FCSHP. Tara Kiran, MD, MSc, CCFP, FCFP. Kori Leblanc, BScPhm, PharmD, ACPR. Catia Marzolini, PharmD, PhD. Kate Miller, MD, CCFP, FCFP. Menaka Pai, MSc, MD, FRCPC. Adrian Poon, BA. Alice Tseng, PharmD, FCSHP, AAHIVP. Kristen Watt, BScPhm, RPh. Samira Jeimy, MD, PhD, FRCPC. Reviewed by: Rosemary Killeen, BScPhm, PGCert, RPh.

For questions about the reproduction, adaptation, translation, or other uses of this material, contact: phrcpd@uwaterloo.ca

Financial contribution from:
Public Health Agency of Canada
NSERC CRSNG
